# Closing the gap between ^19^F and ^18^F chemistry

**DOI:** 10.1186/s41181-021-00143-y

**Published:** 2021-09-25

**Authors:** Javier Ajenjo, Gianluca Destro, Bart Cornelissen, Véronique Gouverneur

**Affiliations:** 1 Medical Research Council Oxford Institute for Radiation Oncology, University of Oxford, Oxford, OX3 7DQ UK; 2 Chemistry Research Laboratory, Department of Chemistry, University of Oxford, Oxford, OX1 3TA UK

**Keywords:** Positron emission tomography, Radiochemistry, Radiofluorination, Fluoride, Fluorine

## Abstract

Positron emission tomography (PET) has become an invaluable tool for drug discovery and diagnosis. The positron-emitting radionuclide fluorine-18 is frequently used in PET radiopharmaceuticals due to its advantageous characteristics; hence, methods streamlining access to ^18^F-labelled radiotracers can make a direct impact in medicine. For many years, access to ^18^F-labelled radiotracers was limited by the paucity of methodologies available, and the poor diversity of precursors amenable to ^18^F-incorporation. During the last two decades, ^18^F-radiochemistry has progressed at a fast pace with the appearance of numerous methodologies for late-stage ^18^F-incorporation onto complex molecules from a range of readily available precursors including those that do not require pre-functionalisation. Key to these advances is the inclusion of new activation modes to facilitate ^18^F-incorporation. Specifically, new advances in late-stage ^19^F-fluorination under transition metal catalysis, photoredox catalysis, and organocatalysis combined with the availability of novel ^18^F-labelled fluorination reagents have enabled the invention of novel processes for ^18^F-incorporation onto complex (bio)molecules. This review describes these major breakthroughs with a focus on methodologies for C–^18^F bond formation. This reinvigorated interest in ^18^F-radiochemistry that we have witnessed in recent years has made a direct impact on ^19^F-chemistry with many laboratories refocusing their efforts on the development of methods using nucleophilic fluoride instead of fluorination reagents derived from molecular fluorine gas.

## Introduction

Positron emission tomography (PET) is a highly sensitive imaging technique that enables non-invasive in vivo characterisation of biochemical processes at a molecular level. It is an invaluable tool for drug discovery, diagnosis, therapeutic assessment, and patient stratification, thereby facilitating the treatment of numerous diseases (Shields et al. 1998). This imaging technology relies on the design and preparation of radiolabelled probes capable of providing quantitative and qualitative information on metabolic processes as well as receptor-ligand interactions. In this context, [^18^F]fluorodeoxyglucose ([^18^F]FDG) used routinely in the clinic has dominated the field for more than 40 years (Bombardieri et al. 2001). Over the past decade, the interest in ^18^F-labelled organic molecules for PET studies has increased significantly in part due to the appearance of innovative methods for late-stage ^19^F-fluorination, and the key role of fluorine-containing compounds in medicinal chemistry (Almuhaideb et al. 2011).

For PET imaging, ^18^F is a commonly used radioisotope due to its advantageous nuclear and physical characteristics. The radioisotope ^18^F decays to ^18^O by positron emission (β^+^ 97%), it has relatively low positron energy (0.635 MeV), a half-life of 109.8 min, and a short positron linear range in tissue (max 2.3 mm) (Jacobson et al. 2015), ^18^F-Radiopharmaceuticals can also be transported and therefore administered in PET facilities that are not equipped with a cyclotron on site. Ideally, ^18^F-radiotracers must be synthesised in high molar activity (A_m_) which is defined as the measured radioactivity per mole of compound, and expressed in Bq/mol or GBq/μmol) (Alauddin 2002). This criterion is important when imaging low tissue density targets such as neuroreceptors, to facilitate PET microdosing studies, and to extend the distance-time over which radiotracers can be transported for scanning. ^18^F is produced by the nuclear reaction ^18^O(*p*,*n*)^18^F (Jacobson and Chen 2010). The most common protocol involves proton bombardment of ^18^O-enriched water to afford an aqueous solution of [^18^F]F^−^. Alternatively, carrier-added gaseous [^18^F]F_2_ can be obtained from the gas target ^18^O_2_ (Bergman and Solin 1997). Aqueous [^18^F]F^−^ is preferred over [^18^F]F_2_ as it is routinely available in high molar activity A_m_ (typically 40–400 GBq/µmol) from medical cyclotrons (10–18 MeV). For most reactions, aqueous [^18^F]F^−^ requires desolvation to enhance its nucleophilicity, which is achieved by trapping ^18^F^−^ on an ion-exchange resin followed by elution with an aqueous solution of base and a phase transfer agent (PTA), and finally azeotropic drying (Cole et al. 2014). In some cases, the presence of base and phase transfer agent may impact detrimentally the radiolabelling step or post-labelling transformations; if this is the case, alternative elution processes or additional purification may be required. This review, which focuses on methods for thermodynamically favourable but kinetically challenging C–^18^F bond formation, will discuss the interplay between ^19^F-chemistry. ^18^F-radiochemistry for the labelling of (bio)molecules, and illustrate how the advances made in the last decades have streamlined access to radiotracers of increasing structural complexity.

For many years, methods for C–^18^F bond formation were largely limited to nucleophilic aliphatic substitution (S_N_2) and nucleophilic aromatic substitution (S_N_Ar) processes using [^18^F]F^−^ (Schirrmacher et al. 2007). These reactions are fundamental to PET imaging by enabling access to [^18^F]FDG and a range of suitably activated [^18^F]fluoro(hetero)arenes. With the development of late-stage ^19^F-fluorination methodologies, the opportunity to invigorate ^18^F-radiochemistry arose, even if not without challenges. Firstly, fluorination (^19^F) methodologies use a range of commercially available nucleophilic, electrophilic, and radical fluorine sources that are not available as ^18^F-isotopologues (Brooks et al. 2015). Secondly, ^18^F-radiochemistry must consider radioisotope decay, radiolysis, and unusual stoichiometry that can impact reaction kinetics and outcome. Indeed, ^18^F-radiochemistry employs a large excess of non-radioactive precursor (μmol–mmol) relative to the ^18^F-source (pmol–nmol), which can also lead to time-consuming purification (Cai et al. 2008). Thirdly, the production of ^18^F-labelled radiopharmaceuticals for clinical usage must comply with current good manufacturing practice (cGMP) regulations to ensure the quality and safety criteria according to pharmacopoeia specifications (Petroni et al. 2012). This review will summarise the progress made to date, and will be broadly divided into ^18^F-fluorination reactions using reagents derived from [^18^F]F_2_, and [^18^F]F^−^. Methods to prepare [^18^F]perfluoroalkylated radiotracers are discussed next, as well as the specific challenges associated with the ^18^F-labelling of biomolecules via C–^18^F bond formation.

### ^18^F-Fluorination with [^18^F]F_2_ and [^18^F]F_2_-derived reagents

**Fig. 1:**
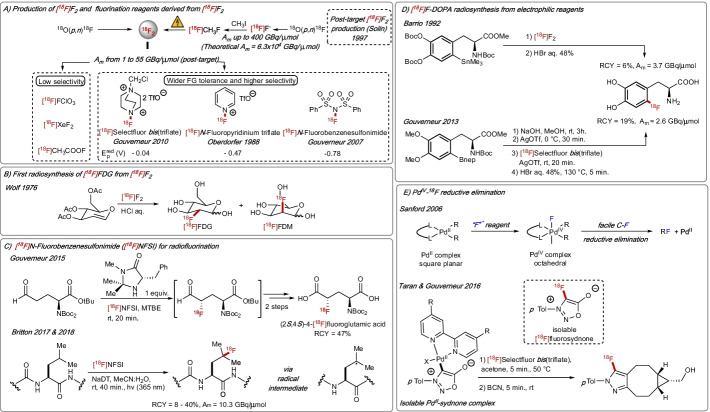
**A** Production of [^18^F]F_2_ and fluorination reagents derived from [^18^F]F_2_. **B** Radiosynthesis of [^18^F]FDG from [^18^F]F_2_. **C** Radiolabelling of aminoacids and peptides with [^18^F]NFSI. **D** Radiosynthesis of [^18^F]FDOPA. **E** Radiofluorination of [Pd^II^]-sydnone complex. MTBE = methyl *tert*-butyl ether. NaDT = sodium decatungstate. Bnep = boronate neopentylglycol ester. BCN = bicyclo[6.1.0]nonyne

Advances in late-stage functionalisation of complex molecules for the incorporation of fluorine (^19^F) have heavily relied on the availability of commercially available fluorination reagents derived from F_2_ gas (Rozatian and Hodgson 2021). Today, cyclotron-produced [^18^F]F_2_ gas remains the parent reagent for the majority of electrophilic and radical reactions for C–^18^F bond formation. The nuclear reactions ^20^Ne(d,α)^18^F or ^18^O(*p*,*n*)^18^F are applied to access [^18^F]F_2_ with A_m_ in the range of 0.04–0.40 GBq/µmol and 0.35–2.00 GBq/µmol, respectively (Bishop et al. 1996). The process requires the addition of carrier F_2_ gas, therefore [^18^F]F_2_ leads to labelled molecules with molar activity significantly lower than those obtained from cyclotron-produced ^18^F-fluoride. Also, this radiochemistry is inherently limited to a maximum theoretical radiochemical yield (RCY) of 50%. Major improvements in the synthesis of [^18^F]F_2_ were made by Solin and co-workers by subjecting a mixture of [^18^F]CH_3_F (formed by reacting [^18^F]F^−^ with CH_3_I) and F_2_ (carrier gas) to high voltage electrical discharge, a process involving atomisation as confirmed by optical emission spectrometry (Forsback et al. 2008). This protocol affords [^18^F]F_2_ in significantly higher molar activity (A_m_ up to 55 GBq/µmol) (Fig. 1A). Further advances involve the use of the replacement of F_2_ with carrier gas sulfur hexafluoride (SF_6_) (Krzyczmonik et al. 2017).

Despite its high reactivity, [^18^F]F_2_ has been successfully employed for the synthesis of [^18^F]FDG (Fig. 1B) (Fowler and Ido 2002), 3,4-dihydroxy-6-[^18^F]fluoro-L-phenylalanine (6-[^18^F]FDOPA) (Forsback et al. 2012), and 2-(2-nitro-1*H*-imidazol-1-yl)-*N*-(2,2,3,3,3-[^18^F]pentafluoropropyl)-acetamide ([^18^F]EF5) (Eskola et al. 2012), a radiotracer for which no route from [^18^F]fluoride has been reported to date. Numerous electrophilic ^18^F-fluorinating reagents were prepared from [^18^F]F_2_ including at first instance [^18^F]perchloryl fluoride ([^18^F]FClO_3_) (Hiller et al. 2018), acetyl [^18^F]hypofluorite ([^18^F]AcOF) (Adam et al. 1986) and [^18^F]XeF_2_, (Chirakal et al. 1984) the latter accessible from the reaction of [^18^F]F_2_ and Xe at 390 °C. In this series, [^18^F]XeF_2_ stands out because Pike reported that this reagent is also accessible by isotopic exchange of XeF_2_ with [^18^F]F^−^ (Lu et al. 2010). These advances enabled the regioselective fluorination of aryl lithium precursors with [^18^F]FClO_3_ (Hiller et al. 2008), and the synthesis of 6-[^18^F]FDOPA from either [^18^F]AcOF or [^18^F]XeF_2_ (Adam et al. 1986; Firnau et al. 1980), albeit with poor regioselectivity and low RCYs. A major departure in electrophilic ^18^F-fluorination resulted from the ^18^F-labelling of *N*-F fluorination reagents featuring a spectrum of oxidising power and reactivity; these reagents all prepared from [^18^F]F_2_ include [^18^F]*N*-fluoropyridinium ([^18^F]NFP) as reported by Oberdorfer (Oberdorfer et al. 1988), as well as [^18^F]*N*-fluorobenzenesulfonimide ([^18^F]NFSI) (Teare et al. 2007) and 1-chloromethyl-4-[^18^F]fluoro-1,4-diazoniabicyclo-[2.2.2]octane *bis*(triflate) ([^18^F]Selectfluor *bis*(triflate)) (Teare et al. 2010), two reagents prepared by Gouverneur, Luthra and Solin. With these tamed “[^18^F]*N*–F” reagents in hand, new ^18^F-radiochemical transformations became possible. The synthesis of diethyl 2-[^18^F]fluoro-2-phenylmalonate was accomplished in 1988 by Maier-Borst employing [^18^F]*N*-fluoropyridinium triflate (Oberdorfer et al. 1988). Twenty years later, Luthra and Gouverneur reported the suitability of [^18^F]NFSI for the synthesis of [^18^F]α-fluoroketones by fluorodesilylation of silyl enol ethers (Teare et al. 2007), and the enantioselective α-^18^F-fluorination of aldehydes (Buckingham et al. 2015a), a method applicable to access enantioenriched (2*S*,4*S*)-4-[^18^F] fluoroglutamic acid (Fig. 1C). This same [^18^F]F-reagent was used by Britton for the tungstate-mediated site-selective ^18^F-fluorination at branched C–H bonds in amino acids, as exemplified by the synthesis of 4-[^18^F]fluoroleucine in 23% RCY, and the radiofluorination of unprotected leucine-containing tetrapeptides (Fig. 1C) (Nodwell et al. 2017; Yuan et al. 2018). Electrophilic ^18^F-fluorination reagents derived from [^18^F]F_2_ have also granted access to ^18^F-fluoroarenes not accessible upon nucleophilic aromatic substitution with [^18^F]fluoride. Early studies demonstrated the feasibility of electrophilic fluorodemercuration employing [^18^F]AcOF (Chaly et al. 1994), and fluorodesilylation and fluorodestannylation using [^18^F]F_2_ including application to the radiosynthesis of 6-[^18^F]FDOPA (6% RCY, A_m_ = 3.7 GBq/µmol) (Fig. 1D) (Namavari et al. 1992). Gouverneur reported the first example of ^18^F-fluorodeborylation with [^18^F]Selectfluor *bis*(triflate) and AgOTf, a reaction affording 6-[^18^F]FDOPA in 19% RCY and A_m_ of 2.6 GBq/µmol (Fig. 1D) (Stenhagen et al. 2013). More recently, Taran with Gouverneur demonstrated that σ-sydnone Pd^II^ complexes underwent oxidative ^18^F-fluorination with [^18^F]Selectfluor to afford ^18^F-labelled sydnones amenable to ultra-fast click chemistry (Liu et al. 2016). This transformation, building on the discovery of Sanford that reductive elimination from [Pd^IV^–F] is more facile than from [Pd^II^–F] complexes, involves an ^18^F-labelled σ-sydnone Pd^IV^–F complex undergoing reductive elimination for C–^18^F bond formation (Fig. 1E).

Over the years, [^18^F]F_2_-based radiochemistry has proven to be undoubtedly challenging but the studies described in this section have served as a solid foundation for many of the recent advances made in radiochemistry with [^18^F]fluoride. Specifically, the realisation that many reactions are feasible applying oxidative ^18^F-fluorination with [^18^F]F_2_ or its derivatives has prompted the development of reactions combining a [^18^F]fluoride source and an external oxidant. These ^18^F-labelling processes will be described in the next sections. For [^18^F]F^+^-based radiochemistry to keep flourishing, a method that gives access to ^18^F-labelled *N*-F reagents from [^18^F]fluoride in high molar activity and that does not require the handling of gaseous F_2_ or SF_4_ would represent a significant advance in the field.

### ^18^F-Fluorination with [^18^F]fluoride

[^18^F]Fluoride, which is produced as an aqueous solution in enriched [^18^O]H_2_O, is the primary source of ^18^F for the vast majority of methodologies currently available for C–^18^F bond formation. Solvation by water renders [^18^F]fluoride unsuitable for most transformations (Hefter and McLay 1988). This issue is solved by adsorption of aqueous [^18^F]fluoride onto an anion-exchange resin (AEX) column, commonly quaternary methylammonium (QMA) cartridges, subsequently eluted with a small volume of a MeCN-water mixture containing a base (K_2_CO_3_) and a metal-chelating cryptand ligand such as Kryptofix® (K_2.2.2._) serving as phase transfer agent (PTA), followed by azeotropic drying (Mossine et al. 2017). The resulting [^18^F]F^−^ residue is then dissolved in a polar aprotic solvent for ^18^F-labelling. With this protocol, the use of anion-exchange cartridges also defines the nature of [^18^F]F^−^ counter-ion, and enhances the purity of [^18^F]F^−^ by removing impurities arising from its production and radiolysis, such as radicals and metal ions. The process has been automated on radiosynthesis modules for routine use in research and clinical environments. For specific ^18^F-radiolabelling reactions, new elution protocols for [^18^F]fluoride were developed and will be described when necessary. Notably, some radiofluorination processes conducted in aqueous media or polar protic solvents have been reported, perhaps more strikingly nucleophilic ^18^F-fluorination (S_N_2) carried out in the presence of the fluorinase enzyme (O’Hagan et al. 2002).

#### ***Csp***^***2***^***–F bond formation***

**Fig. 2:**
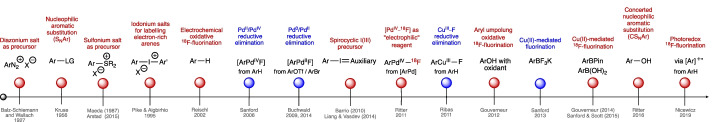
Timeline for (hetero)aryl–^19^F/^18^F bond formation from [^18^F]fluoride. FG = functional group. R = EWG or EDG. EWG = electron-withdrawing group. EDG = electron-donating group. X = counter-anion. LG = leaving group

Metabolically robust fluorine-containing (hetero)arenes are prevalent motifs in pharmaceutical drugs and PET radiotracers (Kuchar and Mamat 2015). As a result, the development of methods for ^19^F– and ^18^F–Csp^2^ bond formation has become a very active field of organic chemistry. Although a plethora of reactions are available for ^19^F-fluorination (Balz and Schiemann 1927), methods for ^18^F–Csp^2^ formation using [^18^F]F^−^ have appeared at a slower pace. Figure 2 illustrates the interplay between ^19^F- and ^18^F-chemistry for the synthesis of fluoro(hetero)arenes with a timeline highlighting some of the key conceptual advances established to date (Fig. 2).

#### *Metal-free Csp*^*2*^*–F bond formation*

**Fig. 3:**
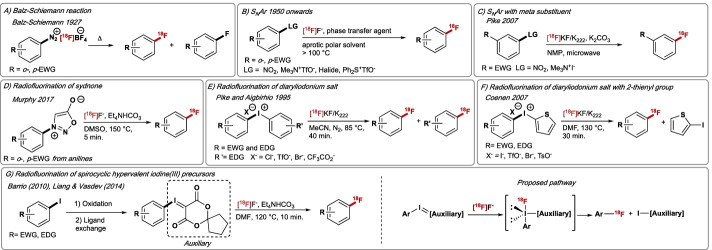
**A** Balz-Schiemann reaction. **B** Nucleophilic aromatic substitution with [^18^F]F^−^. **C** Microwave-assisted radiofluorination of *m*-substituted arene precursors. **D** Radiofluorination of sydnones. **E** Reactions of [^18^F]F^−^ with diaryliodonium salts affording electron-rich aryl [^18^F]fluoride. **F** Reactions of [^18^F]F^−^ with diaryliodonium salts bearing 2-thienyl group. **G** Radiofluorination of spirocyclic hypervalent iodine(III) precursors for non-activated and hindered arenes. EWG = electron-withdrawing group. EDG = electron-donating group. LG = leaving group

The first synthesis of electron-rich and neutral [^18^F]fluoroarenes was reported in 1927 applying the Balz-Schiemann reaction (Fig. 3A) (Balz and Schiemann 1927). Thermal fluorodediazonation of aryl diazonium [^18^F]tetrafluoroborate was successful but afforded [^18^F]fluoroarenes with low A_m_ due to the use of [^18^F]BF_4_^−^ as counter-anion and low RCYs; diazonium with other counterions (e.g., sulfonate) gave superior results (Knochel and Zwernemann 1996). This methodology was applied to [^18^F]fluorophenylalanine, [^18^F]fluorotryptophan, and 5-[^18^F]FDOPA (Argentini et al. 1994). The related Wallach reaction also allowed access to [^18^F]fluoroarenes in the presence of [^18^F]F^−^ by thermal decomposition of aryldiazonium salts formed in situ from triazines; [^18^F]Spiperone and [^18^F]fluoro-α-methylphenylalanine were prepared applying this methodology (Pages et al. 2001). In the mid-1950s, nucleophilic aromatic substitution (S_N_Ar) became the method of choice to incorporate [^18^F]fluoride into activated electron-deficient (hetero)arenes (Finger and Kruse 1956). In this process, a pre-installed leaving group such as NO_2_, NR_3_^+^ (R = alkyl) or halogen (F, Cl, Br, I) is displaced with [^18^F]F^−^ (Preshlock et al. 2016a). These reactions are carried out at high temperatures in polar aprotic solvents and are restricted to arenes featuring an electron-withdrawing substituent (e.g., NO_2_, CF_3_, CN) positioned *ortho* or *para* relative to the leaving group (Fig. 3B). Substitution of halides with [^18^F]F^−^ (Halex) is typically less efficient than substitution of NO_2_ or NR_3_^+^. As expected, S_N_Ar of the fluoro(hetero)arenes with [^18^F]F^−^ (^19^F/^18^F Halex) gives low molar activity. Decades later, Pike reported the microwave-assisted (90 W, 150 °C) ^18^F-radiolabelling of arenes bearing electron-withdrawing groups positioned *meta* relative to the leaving group; this advance is significant as the resulting [^18^F]fluoroarenes are inaccessible under conventional S_N_Ar conditions (Fig. 3C) (Lazarova et al. 2007). More recently, Murphy employed sydnones, that are themselves prepared from anilines, as an alternative leaving group for ^18^F-fluorination via S_N_Ar (Narayanam et al. 2017). The method is compatible with electron-deficient arenes (Fig. 3D). Inspired by the pioneering work from Grushin on nucleophilic aromatic substitution of diaryliodonium salts (Grushin et al. 1992), Pike and Aigbirhio adapted this methodology to access electron-rich and -deficient [^18^F]fluoroarenes (Fig. 3E) (Pike and Aigbirhio 1955). *p*-[^18^F]Fluoroanisole and *p*-[^18^F]fluorotoluene that are inaccessible by S_N_Ar were successfully radiolabelled in high RCYs. Nucleophilic [^18^F]F^−^ addition on unsymmetrical diaryliodonium salts occurred at the most electron-deficient ring, therefore electron-rich substrates required electronic tuning of the ancillary ring to avoid undesired competitive *ipso*-substitution (Yamada et al. 1974). Specifically, Coenen and co-workers reported that 2-thienyliodinium salts are well suited for the radiofluorination of electron-rich arenes (Fig. 3F) (Ross et al. 2007). In 2014, based on pioneering work disclosed by Barrio (Satyamurthy and Barrio 2010). Liang and Vasdev described that spirocyclic hypervalent iodine(III) precursors are also amenable to nucleophilic ^18^F-fluorination with [^18^F]fluoride (Fig. 3G) (Rotstein et al. 2014). This methodology enabled the radiofluorination of a large range of electronically and sterically demanding arenes, often with improved results in comparison to diaryl iodonium salt precursors. This methodology was employed to automate the radiosynthesis of [^18^F]FPEB (non-decay corrected (ndc) 20% RCY, A_m_ = 666 GBq/μmol) (Liang et al. 2019). The necessity of tailored precursors (some unstable or challenging to prepare) to match the steric and electronic profile of the radiotracer under investigation reamins a hurdle to overcome when applying this methodology.

**Fig. 4:**
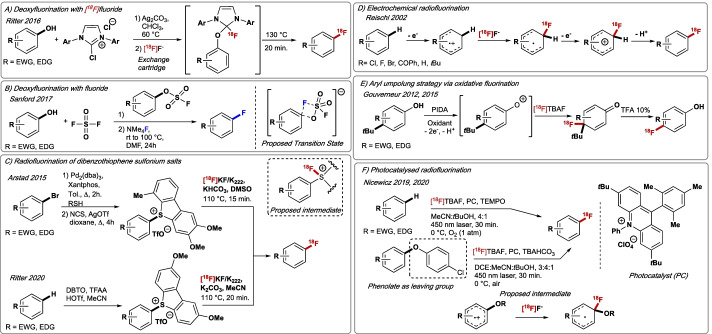
**A** Concerted nucleophilic aromatic substitution (CS_N_Ar) of phenols with [^18^F]F^−^. **B** CS_N_Ar via aryl fluorosulfonate intermediates. **C** Dibenzothiophene sulfoniums as leaving groups for aromatic ^18^F-fluorination. **D** Electrochemical radiofluorination. **E** Aryl umpolung strategy with the use of external oxidant. **F** Photoredox radiofluorination. EWG = electron-withdrawing group. EDG = electron-donating group. NCS = *N*-Chlorosuccinimide. DBTO = dibenzothiophene *S*-oxide. TFAA = trifluoroacetic anhydride. TfOH = triflic acid. PIDA = phenyliodine diacetate. PC = photocatalyst

Up to this point, most molecules subjected to labelling require pre-functionalisation for ^18^F-incorporation. Methods that would displace commonly found functionalities such as alcohols or carboxylic acids with [^18^F]fluoride would present a significant advance, with site-selective C–H ^18^F-fluorination (Szpera et al. 2020) standing out as a pathway to accelerate radiotracers development. In 2016, Ritter and co-workers reported the PhenoFluor-mediated ^19^F. F/^18^F-deoxyfluorination of phenols (Neumann et al. 2016). The process involves the formation of activated uronium intermediates followed by nucleophilic displacement with [^19^F/^18^F]F^−^ via concerted nucleophilic aromatic substitution (CS_N_Ar). In contrast to conventional S_N_Ar, CS_N_Ar avoids the formation of high-energy intermediates, thereby widening the scope of (hetero)arene substrates (Fig. 4A). The method benefits from easy access to uronium intermediates upon reaction of phenols with chloroimidazolium chloride in the presence of a mild base (Ag_2_CO_3_), and does not require anhydrous or air-free condition at any stage of the (radio)synthesis. Advantageously, uronium intermediates can be used to elute [^18^F]fluoride directly from the anion-exchange cartridge. This operationally simple protocol is suitable for ^18^F-labelling electron-rich and electron-deficient (hetero)arenes, and displays broad functional group tolerance including amines, thioethers and amides. An alternative nucleophilic deoxyfluorination of phenols was developed by Sanford in 2017 (Fig. 4B) (Schimler et al. 2017). The reaction proceeds via displacement of in situ formed aryl sulfurofluoridate with tetramethylammonium fluoride (NMe_4_F). The translation of this methodology to ^18^F-radiochemistry, not reported to date, will likely be limited by ^18^F-fluoride leaching, which will impact detrimentally on molar activity. The use of sulfonium salts as precursors for radiolabelling has also been explored. Reports of Maeda on the reaction of dimethylarylsulfonium salts with [^18^F]TBAF (*n*-[^18^F]tetrabutylammonium fluoride) for aryl–^18^F bond formation date back to 1987 (Meada et al. 1987). The process lacks practicality as it is highly limited to electron-deficient aryl systems, and the observation of competing demethylation releasing volatile [^18^F]CH_3_F as the predominant pathway. More recently, the process was vastly improved by Arstad who reported the ^18^F-fluorination of dibenzothiophene sulfonium salts to access [^18^F]fluoro(hetero)arenes of various electronic profiles (Fig. 4C) (Gendron et al. 2018). Clinically relevant 3-[^18^F]fluorodeprenyl, [^18^F]FPEB, and [^18^F]P3BZA were successfully ^18^F-labelled in 32%, 55% and 52% RCY, respectively (Gendron et al. 2018). This method requires tailoring of the electronic profile of the triaryl sulfonium precursors for selective *ipso*-^18^F-incorporation at the desired aryl group. Building on this study, Ritter reported in 2020 further improvement of this radiochemistry with a late stage C–H dibenzothiophenylation of (hetero)arenes followed by [^18^F]fluorination, a process that avoids the multi-step synthesis of the triarylsulfonium precursors (Fig. 4C) (Xu et al. 2020). This radiofluorination of dibenzothiophene sulfonium salts benefits from direct [^18^F]F^−^ elution from the anion-exchange cartridge by the sulfonium salt precursor itself.

Oxidative electrochemical fluorinations were pioneered by Simons and Harland 1949; the process consists of anodic oxidation of a substrate dissolved in anhydrous liquid HF at a nickel electrode of an electrochemical cell (single electron transfer, SET) to afford an arene radical cation applying an electrical current (voltage) (Simons 1949). Upon trapping with F^−^, this radical cation is converted into fluoroarene. Electrochemical reactions are often conducted at room temperature under atmospheric pressure, and do not require an external oxidant or reductant, thereby minimising waste production. Despite these advantages, the main limitation of these methods is poor selectivity. To address this drawback, selective electrochemical fluorination methodologies featuring control of oxidation potential, molten salts, organic redox mediators (e.g., triarylamines, PhIF_2_), ionic liquids or/and anhydrous aprotic solvents were developed (Sawamura et al. 2012). Building on seminal studies by Rozhkov (Rozhkov and Alyev 1975), Knunyants (Knunyants et al. 1970) and Fuchigami (Narizuka and Fuchigami 1993) electrochemical ^18^F-fluorination was first reported by Reischl (Reischl et al. 2002). Benzene was subjected to C–H oxidative ^18^F-fluorination with [^18^F]fluoride eluted from a QMA cartridge with Et_3_N·3HF or Et_3_N·3HCl/CH_3_CN electrolyte solutions, at 0 °C applying a potential of 2.0 V. [^18^F]Fluorobenzene was produced in 15.6% RCY (A_m_ = 27 GBq/$\mu$mol) when using non-carrier added electrolyte solutions (Et_3_N·3HCl/CH_3_CN). A year later, the same group extended the applicability of this method to variously substituted arenes (Ar–R, R = F, Cl, Br, *t*Bu, COPh) with efficiency and regioselectivity found to be highly dependent on substitution due to electronics (+/–I). As expected, electron-rich arenes that are easier to oxidise gave the best results (Fig. 4D). In 2005, phenylalanine ^18^F-labelled derivatives were produced by anodic oxidation employing Et_3_N·3HF as electrolyte and a potential of 1.5–2 V (Kienzle et al. 2005). Specifically, *N*-[^18^F]trifluoroacetylphenylalanine methyl ester was obtained as a 5:1:4 (*o*:*m*:*p*) mixture of regioisomers in 10.5% RCY (A_m_ = 1.2 GBq/$\mu$mol). Electrochemical radiofluorination of *bis*-Boc-protected 4-*tert*-butylcatechol was reported in 2014 by Sadeghi affording bis-Boc-protected 4-[^18^F]fluorocatechol in 8.9% decay-corrected (dc) RCY (A_m_ = 0.043 GBq/$\mu$mol) (He et al. 2001). The same group disclosed the automated electrochemical radiosynthesis of [^18^F]Celecoxib (2% dc RCY, A_m_ = 0.111 GBq/$\mu$mol) (Lebedev et al. 2017).

Concurrent to these discoveries, non-electrochemical oxidative ^18^F-labelling strategies have emerged. In 2012, Gouverneur studied the oxidative ^18^F-fluorination of unprotected *para*-*tert*-butyl-substituted phenols for the radiosynthesis of 4-[^18^F]fluorophenols (Fig. 4E) (Gao et al. 2012). The proposed mechanism involves two-electron oxidation of phenol followed by [^18^F]fluoride addition. Optimised conditions employ [^18^F]TBAF/TFA in the presence of bis(acetoxy)iodobenzene in dichloromethane. The method tolerates variously *ortho-* and *meta*-substituted phenols affording the desired 4-[^18^F]fluorophenols with A_m_ up to 420 GBq/μmol. In 2015, the same group successfully extended this methodology to the direct C–H oxidative ^18^F-fluorination of aryl sulphonamides (Buckingham et al. 2015b). In 2019, Nicewicz and Li reported an alternative strategy based on photocatalytic ^18^F-fluorination of Csp^2^–H bonds (Fig. 4E) (Chen et al. 2019). The process involves an acridinium-based photocatalyst in the presence of [^18^F]TBAF in MeCN/*t*BuOH under oxygen atmosphere and 450 nm laser irradiation. This method is suitable for C–H radiofluorination of electron-neutral and electron-rich (hetero)arenes in RYCs up to 50%, as exemplified by the ^18^F-labelling of 6-[^18^F]FDOPA (12% RCY). The same group improved this methodology by swapping laser irradiation with blue LED, and suppressing the need for O_2_ bubbling through the reaction mixture by adding *tert*-butyl peroxyacetate (TBPA) (Wang et al. 2020). One year later, Nicewicz and Li further expanded the applicability of this concept with the development of a photoredox-catalysed cation-radical-accelerated S_N_Ar (CRA-S_N_Ar) amination and cyanation of methoxy and benzyloxy nucleofuges (Douglas and Nicewicz 2019) CRA-S_N_Ar also enables selective radiofluorination of electron-rich arenes with [^18^F]TBAF under mild conditions and short reaction times. The strategy afforded 5-[^18^F]fluorouracil ([^18^F]FU) in 82% dc RCY and A_m_ of 74.7 GBq/$\mu$mol.(Tay et al. 2020).

#### Metal-mediated/catalysed Csp^2^–F bond formation

In recent years, transition metal-catalysed methodologies have also emerged as an attractive route for the fluorination of arenes with nucleophilic fluoride sources (Preshlock et al. 2016a).

#### Palladium and nickel

**Fig. 5:**
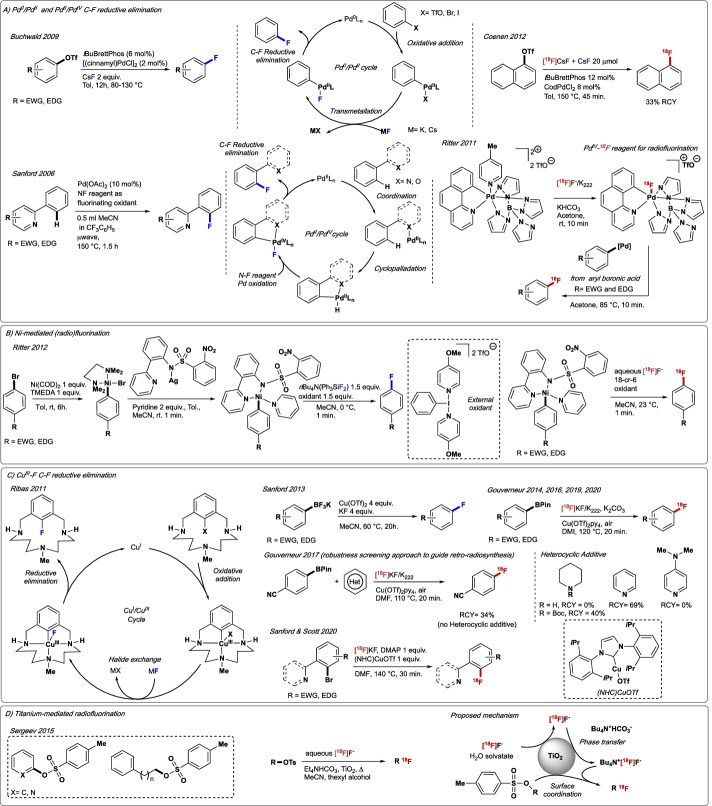
**A** Csp^2^–F bond formation through Pd^0^/Pd^II^ and Pd^II^/Pd^IV^ catalytic cycles and application to radiofluorination. **B** Use of Ni-complex for radiofluorination. **C** Cu^III^–F C–F reductive elimination and application to radiofluorination. **D** Titanium-mediated radiofluorination of aryl and alkyl tosylate derivatives. NHC = *N*-heterocycle carbene. EWG = electron-withdrawing group. EDG = electron-donating group

In 2009, Buchwald reported the first Pd(0)-catalysed fluorination of aryl triflates (Fig. 5A). The use of electron-rich biaryl monophosphine ligands was crucial to favour reductive elimination at [ArPd(II)F] and preventing the formation of unreactive dimeric [LPdAr(F)]_2_ species (Watson et al. 2009). Despite its scope and functional group tolerance, substrates bearing Lewis basic groups such as amines failed to undergo fluorination. A study by Coenen on the applicability of this methodology to ^18^F-labelling led to the preparation of [^18^F]fluoronapthalene in 33% RCY (Fig. 5A) (Cardinale et al. 2012). The necessity for carrier-added CsF for the reaction to proceed, and the generation of reduced side-products that are difficult to separate from the ^18^F-labelled fluoroarenes limit the value of this methodology. Complementing this approach, Sanford reported a Pd(II)-catalysed quinoline/pyridine-directed C–H fluorination of arenes involving reductive elimination at [ArPd(IV)F], an intermediate generated with the fluorination oxidant *N-*fluoro-2,4,6-trimethylpyridinium tetrafluoroborate (Fig. 5A) (Hull et al. 2006). This seminal study inspired the development of numerous metal-catalysed fluorination reactions harnessing the favourable reactivity of high oxidation state metal complexes (Boursalian and Ritter 2018). In 2011, Ritter succeeded in generating a high valent [Pd(IV)^18^F] complex from [^18^F]F^−^, which was shown to react with pre-formed ArPd(II)X themselves formed from aryl boronic acids, to generate [^18^F]fluoroarenes (Lee et al. 2011). The method afforded [^18^F]paroxetine and [^18^F]5-HT_2C_, but suffered from the requirement to prepare structurally Pd-complex species that require know-how expertise in organometallic chemistry (Fig. 5A). The same group developed a one-step radiofluorination of pre-formed [ArNi(II)Br] complexes in the presence of a bespoke external oxidant in a mixture of aqueous [^18^F]fluoride/MeCN (Fig. 5B) (Lee et al. 2012). This oxidative ^18^F-fluorination benefits from short reaction times and the use of precursors accessible by oxidative addition of aryl halide to a Ni(0)complex in the presence of TMEDA, followed by ligand exchange with (2-(2-pyridinyl)phenyl-2-nitrobenzenesulfonamide)silver(I). Further optimisation of this process by Neumaier afforded 6-[^18^F]FDOPA and 6-[^18^F]fluorodopamine (6-[^18^F]FDA) in 7% RCY (A_m_ = 175 GBq/$\mu$mol) and 12% RCY (A_m_ = 60 GBq/$\mu$mol), respectively (Zlatopolskiy et al. 2015).

*III.1.2.2. Copper* To date, copper-mediated Csp^2^–^18^F bond formation is one of the metal-mediated reactions that has made the greatest impact on ^18^F-radiochemistry in recent years. Building on foundational studies on Cu(I)-mediated fluorination of aryl halides by Ribas (Casitas et al. 2011) and Hartwig (Fier and Hartwig 2012) Sanford reported in 2013 the fluorination of aryl boron reagents using high excess of KF and Cu(OTf)_2_ (Tredwell et al. 2014). The proposed mechanism involves the formation of [ArCu(III)F] undergoing C–F reductive elimination to afford fluoroarenes (Fig. 5C). In 2014, Gouverneur successfully adapted this methodology to ^18^F-radiochemistry with the ^18^F-fluorination of bench-stable pinacol-derived (hetero)arylboronic esters in the presence of commercially available Cu(OTf)_2_(py)_4_ (Fig. 5C) (Tredwell et al. 2014). The procedure displays a wide substrate scope including electron-rich, electron-deficient, and sterically hindered aryl boron reagents and broad functional tolerance to afford aryl [^18^F]fluorides in a short time and RCYs up to 83%. Gouverneur reported the application of this method to clinically relevant tracers including 3-[^18^F]fluoro-5-((2-methylthiazol-4-yl)ethynyl)benzonitrile ([^18^F]FMTEB), 3-[^18^F]fluoro-5-(pyridin-2-ylethynyl)benzonitrile ([^18^F]FPEB), [^18^F]flumazenil, *N*-(2,5-dimethoxybenzyl)-*N*-(5-[^18^F]fluoro-2-phenoxyphenyl)acetamide ([^18^F]DAA1106), *m*-[^18^F]fluorobenzylguanidine ([^18^F]MFBG), [^18^F]FDOPA, 6-[^18^F]fluoro-L-*m*-tyrosine ([^18^F]FMT) and [^18^F]FDA (Preshlock et al. 2016b) as well as an extensive study providing radiochemists with guidelines on how to apply this radiochemistry to label complex drug candidates featuring multiple functionalities including nitrogen-containing heterocycles and heteroarenes (Taylor et al. 2017) More recently, Gouverneur also reported the automated radiosynthesis of [^18^F]olaparib, a poly(ADP-ribose) polymerase (PARP) inhibitor in 6% activity yield (AY) and A_m_ = 319 GBq/$\mu$mol, thereby demonstrating the translational power of this versatile Cu-mediated ^18^F-fluorodeboronation (Guibbal et al. 2020). Closely after its disclosure, this method for ^18^F-labelling (hetero)arenes triggered numerous follow-up studies. Sanford and Scott discovered that aryl stannanes and aryl boronic acids are equally suitable for Cu-mediated radiofluorination (Makaravage et al. 2016), and demonstrated value with the automated radiosynthesis of protected 6-[^18^F]fluoro-L-DOPA in 55% RCY. In 2019, these groups developed an improved automated protocol for the synthesis of 6-[^18^F]fluoro-L-DOPA in 4–6% RCY non-decay-corrected (ndc) and A_m_ in the range of 71–140.6 GBq/$\mu$mol, that meets all QC criteria for in-human PET imaging (Mossine et al. 2019, 2020). Further variations of this Cu-mediated methodology make use of alternative precursors including iodonium salts, aryl chlorides and aryl bromides (Ichiishi et al. 2014). In 2021, Scott and Sanford developed a one-pot sequential Ir/Cu-mediated mediated radiofluorination of (hetero)arenes (Wright et al. 2021). The process involves in situ formation of (hetero)aryl boronate esters intermediates via Ir-catalysed *meta*-selective Csp^2^–H borylation that subsequently undergo copper-mediated radiofluorination with [^18^F]TBAF to afford ^18^F-labelled (hetero)aryl in RCYs up to 88%. In addition, based on studies by Liu (Mu et al. 2014), N-heterocyclic carbene (NHC) Cu-complexes were investigated by Sanford and Scott for the ligand-directed radiofluorination of aryl halides (Ar–X, X = Br, Cl, I); the narrow substrate scope and the need of rigorous air-free and anhydrous conditions limit the applicability of this methodology (Fig. 5C) (Sharninghausen et al. 2020).

The value of Cu-mediated ^18^F-fluorination has encouraged the development of novel methods for late-stage nucleophilic ^19^F-fluorination. A recent example is the Cu-catalysed photoinduced decarboxylative fluorination of aryl carboxylic acids reported in 2021 by Ritter (Xu et al. 2021) and by MacMillan (Chen et al. 2021) translation to ^18^F-labelling has not been demonstrated to date. In addition to expanding the range of ^18^F-labelling processes mediated by copper, many efforts have focused on how ^18^F-elution can improve the outcome of these reactions. The use of bases such as K_2_C_2_O_4_, *N*,*N*-dimethylpyridin-4-amine (DMAP) or the development of “low-base” ^18^F-elution protocols have recently been reported (Mossine et al. 2017; Richarz et al. 2014). Also, the addition of alternative ancillary Cu-ligands or the use of different eluents, such as alcohols or solutions of organic bases (e.g., dimethylaminopyridinium trifluoromethanesulfonate (DMAPH^+^TfO^−^), Et_4_N^+^TfO^−^), have resulted in higher RCYs (Mossine et al. 2017). For example, the synthesis of 6-[^18^F]FDOPA employing Bu_4_N^+^TfO^−^ as PTA, and 2-propanol for [^18^F]F^−^ elution, was achieved in 15% RCY and A_m_ = 34–61 GBq/$\mu$mol; the radiosynthesis time was reduced from ~ 120 to 70 min by avoiding the azeotropic drying step (Mossine et al. 2017). This year, a protocol describing the formation of dry [^18^F]TBAF by aliquoting QMA-eluted methanolic solution of [^18^F]fluoride followed by evaporation has contributed to identifying optimal copper-precursor ratio and solvent volume for the synthesis of [^18^F]olaparib (Bowden et al. 2021). This work employs “design of experiments” (DoE), a statistical toolkit that can provide detailed models of processes’ performance with respect to variables, a valuable tool for radiotracer synthesis optimisation.

*III.2.2.3. Titanium* In 2015, van Dam and Sergeev disclosed the ^18^F-fluorination of tosylated arenes with [^18^F]fluoride, TiO_2_ nanoparticles and tetrabutylammonium bicarbonate (TBAB) as phase transfer agent (Sergeev et al. 2015). The authors propose that TiO_2_ is responsible for [^18^F]fluoride desolvation, and promotes ^18^F-fluorination by coordinating the tosylate leaving group. This methodology employs an acetonitrile/thexyl alcohol mixture as the solvent, and tolerates up to 25% v/v of H_2_O. This protocol was suitable for the manual radiosynthesis of [^18^F]fallypride (D2/3R antagonist) in 71% RCY and A_m_ = 185 GBq/$\mu$mol (Fig. 5D).

### Csp^3^–F bond formation

**Fig. 6:**
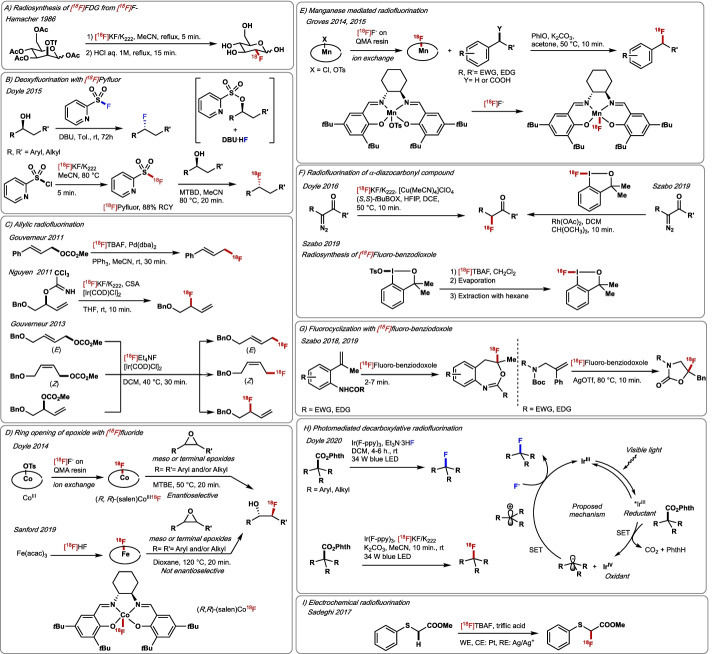
**A** [^18^F]FDG radiosynthesis from [^18^F]fluoride. **B** Radiosynthesis of [^18^F]Pyfluor. **C** Allylic radiofluorination. **D** Ring opening of epoxides by [^18^F]fluoride. **E** Groves Csp^3^–H radiofluorination and ^18^F-fluorodecarboxylation. **F** Radiofluorination of α-diazocarbonyl compound. **G **Oxidative ^18^F-fluorocyclization. **H** Photo-mediated decarboxylative radiofluorination of activated esters. **I** Electrochemical radiofluorination. EWG = electron-withdrawing group. EDG = electron-donating group. PhthH = *N*-Hydroxyphthalimide

Traditional methods for Csp^**3**^–F bond construction are based on the nucleophilic displacement of pre-installed leaving groups such as halide (Cl, Br), tosylate (OTs) or triflate (OTf) with F^−^, and deoxyfluorination reactions (Wu 2014). Most of these processes follow a stereospecific bimolecular substitution pathway (S_N_2) and are limited to the preparation of primary or secondary alkyl fluoride. Fluoride interacts with protic solvents through hydrogen bonding resulting in diminished nucleophilicity (Liang et al. 2017), therefore S_N_2 reactions with alkali fluorides are conducted in polar aprotic solvents such as dimethylformamide (DMF), dimethylsulfoxide (DMSO) or acetonitrile (CH_3_CN); however, cases of aliphatic fluorination in protic solvents have been reported. The most striking example features the natural fluorinase enzyme, discovered by O’Hagan, which is capable of catalysing nucleophilic fluorination with F^−^ in water (Hamacher et al. 1986). Fluoride exhibits enhanced Brønsted basicity under anhydrous conditions, that may result in narrow functional group compatibility and the occurrence of competing side reactions. Specifically, undesired elimination processes that may be significant in radiofluorination due to the unusual stoichiometry of radiochemical reactions, the necessity to apply high temperatures, and the use of basic solutions for [^18^F]fluoride elution. Nucleophilic substitution (S_N_2) with [^18^F]F^−^ is the most common method for the radiosynthesis of ^18^F-labelled alkyl-containing radiopharmaceuticals because [^18^F]F^−^ is readily accessible in high molar activity. In 1986, Hamacher reported the synthesis of [^18^F]FDG from mannose triflate, [^18^F]F^−^ and Kryptofix (K_2.2.2._) as phase transfer agent (Fig. 6A) (Hamacher et al. 1986). The use of ionic liquids (ILs) such as 1-butyl-3-methylimidazolium tetrafluoroborate [emim][BF_4_] or 1-ethyl-3-methylimidazolium triflate [bmim][OTf] increases the efficiency of the process (Kim et al. 2004). The synthesis of [^18^F]FDG and 3′-deoxy-3′-[^18^F]fluorothymidine ([^18^F]FLT) was also accomplished with aqueous [^18^F]F^−^ in the presence of ILs and Cs_2_CO_3_ in 75% and 65% RCYs, respectively (Moon et al. 2006). Numerous radiotracers including 1-(2-nitro-imidazolyl)-3-[^18^F]fluoro-2-propanol ([^18^F]FMISO) (hypoxia) (Kamarainen et al. 2004). *cis*-4-[^18^F]fluoro-L-proline (collagen) (Morgan et al. 2021) and *O*-(2-[^18^F]fluoroethyl)-L-tyrosine (glioma grading) (Wester et al. 1999) were successfully ^18^F-radiolabelled via S_N_2 with [^18^F]fluoride. Deoxyfluorination of aliphatic alcohols is classically accomplished employing diethylaminosulfur trifluoride (DAST) or bis(2-methoxyethyl)aminosulfur trifluoride (Deoxo-Fluor) (Singh and Shreeve 2002). These reagents are unstable, display limited functional group tolerance, and can lead elimination side-products. The synthesis of [^18^F]DAST was reported by Straatmann and Welch in 1977 (Straatmann and Welch 1977). Its application to [^18^F]deoxyfluorination was exemplified with the synthesis of [^18^F]fluoromethane, [^18^F]fluoroethane and 2-[^18^F]fluoroethanol. Second-generation deoxyfluorination reagents have recently appeared that display higher stability and increased molar activity and chemoselectivity. Specifically, pyridine-2-sulfonyl fluoride (PyFluor) (Nielsen et al. 2015) and 1,3-bis(2,6-diisopropylphenyl)-2-fluoroimidazolium tetrafluoroborate (AlkylFluor) (Goldberg et al. 2016) an analogue of 1,3-bis(2,6-diisopropylphenyl)-2,2-difluoro-2,3-dihydro-1H-imidazole (PhenoFluor) were recently reported by Doyle (2015) and Ritter (2016), respectively. PyFluor was ^18^F-labelled by reacting pyridine-2-sulfonyl chloride with [^18^F]KF and used for nucleophilic aliphatic ^18^F-fluorination reactions in the presence of Kryptofix®. This chemistry gave access to 2,3,4,6-tetra-*O*-benzyl-D-glucopyranosyl [^18^F]fluoride in 13% RCY (Fig. 6B) (Nielsen et al. 2015). During the last decade, it is important to note that a plethora of methodologies for Csp^3^–F bond construction based on C–heteroatom and C–H functionalisation were developed employing Selectfluor, NFSI and *N*-fluoropyridinium triflate (Tarantino and Hammond 2020). These are based on C–F bond formation via reductive elimination from Pd(IV)F complexes or radical pathways (Testa et al. 2019). These transformations represent a powerful toolkit for construction of alkyl fluorides, but despite their value, their impact in Csp3 ^18^F-fluorination is hampered by the necessity of using [^18^F]F_2_ for the preparation of [^18^F]Selecfluor, [^18^F]NFSI and [^18^F]*N*-fluoropyridinium triflate.

As for Csp^2^–F bond construction, transition metals have opened new opportunities for the incorporation of fluorine onto alkyl chains using fluoride source. Early studies have focused on allylic nucleophilic fluorination, an area intensively investigated by many research laboratories including Togni (Hintermann et al. 2006), Brown (Hazari et al. 2009), Gouverneur (Hazari et al. 2009; Hollingworth et al. 2011), Doyle (Katcher and Doyle 2010) and Nguyen (Topczewski et al. 2011) and their co-workers. The process requires careful selection of the leaving group, metal, and fluoride source to favour reductive elimination leading to C–F bond formation as opposed to undesired product consumption via C–F bond oxidative addition. In 2011, Gouverneur and co-workers reported the ^18^F-fluorination of allyl *p*-nitrobenzoate substrates with [^18^F]TBAF in the presence of Pd(dba)_2_, a reaction affording ^18^F-labelled allylic fluoride such as (*E*)-(3-[^18^F]fluoroprop-1-en-1-yl)benzene in RCYs up to 52% (Fig. 6C) (Hazari et al. 2009). In the same year, Nguyen reported a successful Ir-catalysed allylic ^18^F-fluorination of trichloroacetimidates with [^18^F]KF in the presence of [Ir(COD)Cl]_2_ and camphorsulfonic acid, a system producing [^18^F]HF in situ (Fig. 6C) (Topczewski et al. 2011). The same group subsequently disclosed that [IrMeO(COD)_2_] performs better than [Ir(COD)Cl]_2_, thereby offering access to a wider range of ^18^F-labelled allyl fluorides with excellent regioselectivity and RCYs up to 37% (Mixdorf et al. 2019). Considering the importance of selectivity, Gouverneur and co-workers reported that the combined use of [Ir(COD)Cl]_2_ and [^18^F]TBAF provide controlled access to ^18^F-labelled branched, linear-*E* or linear-*Z* allylic fluorides in RCYs up to 76% (Fig. 6C) (Benedetto et al. 2013). In 2014, building on the pioneering work of Bruns and Haufe (2000). Doyle reported the opening of racemic epoxides with (*R*,*R*)-[^18^F](salen)Co(III)F under mild conditions to access radiotracers containing a [^18^F]fluorohydrin moiety (Graham et al. 2014). Advantageously, (*R*,*R*)-[^18^F](salen)Co(III)F was prepared by direct elution of [^18^F]F^−^ from the QMA cartridge using air-stable (*R*,*R*)-(salen)CoOTs. This asymmetric ^18^F-fluorination method gave access to 1-(2-nitro-imidazolyl)-3-[^18^F]fluoro-2-propanol ([^18^F]FMISO) in both manual (67% RCY, 90% *ee*) and automated (10.6% RCY, A_m_ = 137 GBq/µmol) mode (Fig. 6D). Sandford and Scott developed a similar strategy, although not enantioselective, based on the opening of sterically hindered epoxides by ^18^F-radiofluorination with [^18^F]FeF species prepared by direct elution of [^18^F]fluoride trapped on an anion-exchange cartridge with Fe(acac)_3_ and a solution of acids (CH_3_CO_2_H, CH_3_SO_3_H) (Verhoog et al. 2019). 5-[^18^F]Fluoro-6-hydroxy-cholesterol (adrenal imaging) was obtained in 22% RCY and a molar activity of 7.8 GBq/µmol (Fig. 6D). In 2014, Hooker and Groves described an impressive oxidative Csp^3^–H fluorination with (Mn(III)(salen)OTs), [^18^F]F^−^ and PhIO (Fig. 6E) (Huang et al. 2014). The method benefits from [^18^F]fluoride elution by the manganese complex, displays wide functional group tolerance, and presents selectivity for benzylic C–H on a range of substrates. For example, [^18^F]celestolide (musk fragrance) was successfully ^18^F-radiolabelled in 10% RCY and A_m_ of 99 GBq/µmol. In 20^18^, the same group adapted this methodology for the ^18^F-fluorination of less activated Csp^3^–H bonds with Mn(TPFPP)OTs. [^18^F]3-Fluorobutyl benzoate was obtained in 39% RCY and A_m_ = 46 GBq/μmol, and various biologically active compounds such as [^18^F]fluorotandospirone (5-HT neurotransmitter) in 30% RCY (A_m_ not reported) and 3-[^18^F]-FACPC, an analogue of ^18^F-FACBC (prostate cancer) (60% RCY, A_m_ not reported) were made accessible without the need to prepare pre-functionalised precursors (Liu et al. 2018). This methodology was extended to Mn-mediated decarboxylative ^18^F-fluorination (Huang et al. 2015). Building on these principles, Carroll applied this methodology for the conversion of difluoromethylated arenes into ^18^F-trifluoromethylated arenes; the method employs Mn(salen)Cl, [^18^F]fluoride, PhIO and AgOTf (Carroll et al. 2015). Using this protocol, 1-bromo-4-[^18^F](trifluoromethyl)benzene was ^18^F-labelled in 44% RCY (A_m_ = 4 GBq/μmol). Copper(I)-catalysed fluorination of α-diazocarbonyl has allowed access to α-fluorocarbonyl derivatives, a process reported by Doyle in 2016 (Fig. 6F) (Gray et al. 2016). The proposed mechanism involves the formation of electrophilic metal carbenoids capable of reacting with in situ generated HF. This operationally simple protocol displays broad functional group compatibility, wide substrate scope, and was successfully translated to ^18^F-labelling with [^18^F]KF-Kryptofix®-HFIP under mild conditions. The radiosynthesis of N5-[^18^F]fluoroacetylornithine (N5-[^18^F]FAO) (ornithine decarboxylase imaging) was accomplished in higher yield (RCY = 39%) compared to S_N_2 with [^18^F]F^−^ (RCY = 8%). Recently, Csp^3^–^18^F bond forming reactions were performed with ^18^F-labelled hypervalent iodine(III) reagents prepared from [^18^F]fluoride (Gonzalez et al. 2018). In _19_F-mode, *p*-TolIF_2_ and fluorobenziodoxole (Kohlhepp and Gulder 2016) are versatile reagents for numerous reactions including fluorocyclization, fluorination-ring-expansion, and asymmetric fluorination reactions. Complementing Doyle’s strategy, Szabo reported the Rh-mediated ^18^F-oxyfluorination of diazoketones and diazoamide derivatives with [^18^F]fluorobenziodoxole, itself prepared from [^18^F]fluoride, affording α-[^18^F]fluoro ethers in molar activities up to 216 GBq/µmol (Fig. 6F) (González et al. 2019). Building on studies by Stuart on the fluorocyclisation of carboxylic acids with fluorobenziodoxole (Geary et al. 2015). Lu and Li reported in 2017 the silver-mediated radiofluorination of unsaturated carbamates with [^18^F]fluorobenziodoxole formed in situ from [^18^F]Bu_4_NF (Yang et al. 2017). This methodology led to ^18^F-fluorinated oxazolidine-2-ones in molar activities up to 34 GBq/µmol. Higher RCYs were achieved when [^18^F]Bu_4_NF was purified by extraction with hexane prior to radiolabelling (Fig. 6G). Pursuing this line of research, Szabo reported also the synthesis of [^18^F]fluoro-benzoxazepines from [^18^F]fluorobenziodoxole in RCYs ~ 10% and high A_m_ (up to 396 GBq/µmol) (Gonzalez et al. 2018). In 2020, Doyle reported a decarboxylative photocatalytic (radio)fluorination of redox active *N*-hydroxyphthalimide esters (Fig. 6H) (Webb et al. 2020). The reaction proceeds by two-electron transfers between the Ir catalyst and redox-active ester substrate to afford a carbocation intermediate that is trapped with [^18^F]fluoride. Primary, secondary, and tertiary fluorides are within reach, the latter products being inaccessible by S_N_2 fluorination. ^18^F-Labelling was performed with [^18^F]KF-Kryptofix® and with Ir(Fppy)_3_ in acetonitrile, and afforded 2-(1fluoroethyl)-6-methoxynaphthalene in 11–49% RCY (A_m_ up to 36 GBq/μmol) as well as 2-((4-[^18^F]fluoro-4-[^18^F]methylpentyl)oxy)-1,4-dimethylbenzene in 9% RCY (A_m_ not reported). Electrochemical ^18^F-radiofluorination was recently achieved for Csp^3^–^18^F bond formation (Fig. 6I). Indeed, Sadeghi reported in 2017 the electrochemical ^18^F-labelling of methyl(phenylthio)acetate under controlled potentiostatic conditions (1.4 V) using [^18^F]TBAF in the presence of triflic acid; the process afforded methyl 2-[^18^F]fluoro-2-(phenylthio)acetate in 7% and avoided the use of hydrogen fluoride salts (e.g. Et_3_N·3HF) (Waldmann et al. 2017). In 2018, this methodology was improved by the use of 2,2,2-trifluoroethanol (TFE) and 2,6-di-*tert*-butyl-4-methylpyridine, at low temperature (– 20 °C) in triflic acid (pH = 3) (Balandeh et al. 2018). This protocol allowed access to [^18^F]methyl 2-fluoro-2-(phenylthio)acetate in 5.7% RCY and A_m_ = 42 GBq/µmol, as well as methyl 2-[^18^F]fluoro-2-(methylthio)acetate and methyl 2-[^18^F]fluoro-2-(ethylthio)acetate in 21% and ^18^% RCY, respectively.

### Methods towards ^18^F-perfluoroalkyl-containing molecules

In recent years, methods for perfluoroalkylation have appeared at a fast pace (Barata-Vallejo et al. 2015) and the benefit of perfluoroalkyl substitution in agrochemical and pharmaceutical drug discovery has been amply demonstrated (Prchalová et al. 2014). The advantageous characteristics of these functional groups have not been harnessed to the same extent in PET ^18^F-radiochemistry for several reasons; firstly, many perfluoroalkylation (^19^F) processes rely on the availability of bespoke reagents which themselves require innovative approaches for ^18^F-labelling, and secondly achieving high molar activity is a challenge as these motifs contain more than one atom of fluorine. This section summarises the progress made to date in ^18^F-labelling of perfluoroalkyl groups and the challenges ahead of us.

#### Radiosynthesis of [^18^F]trifluoromethyl-containing molecules

**Fig. 7:**
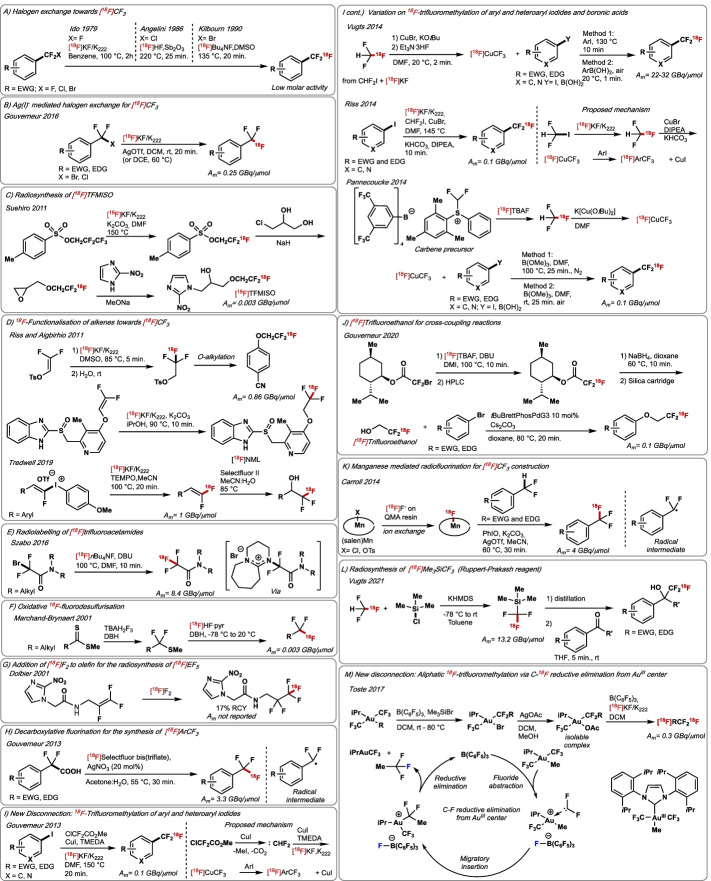
**A** Halogen exchange strategy for [^18^F]CF_3_ construction. **B** Silver-mediated halogen exchange for -CF_3_, -OCF_3_, -SCF_3_, -CF_2_H, -OCF_2_H ^18^F-radiofluorination. **C** Radiosynthesis of [^18^F]TFMISO. **D** ^18^F-Functionalization of fluoroalkenes, **E** Radiofluorination of trifluoroacetamides. **F** Oxidative ^18^F-fluorodesulfurisation. **G** Radiosynthesis of [^18^F]EF5 from [^18^F]F_2_. **H** Decarboxylative radiofluorination for the synthesis of [^18^F]CF_3_. **I** Ar–[^18^F]CF_3_ disconnection: ^18^F-Trifluoromethylation of aryl iodides and aryl boronic acids. **J** [^18^F]Trifluoroethanol for cross-coupling reaction. **K** Manganese-mediated [^18^F]CF_3_ construction. **L** Radiosynthesis of the [^18^F]Me_3_SiCF_3_ ([^18^F]Ruppert-Prakash reagent. **M** New disconnection: Aliphatic ^18^F-trifluoromethylation from C–^18^F reductive elimination from Au^III^ center. EWG = electron-withdrawing group. EDG = electron-donating group

The introduction of the metabolically stable trifluoromethyl group (CF_3_) is key to tune the physicochemical properties of bioactive compounds on demand (Pan 2019) hence, methodologies for the ^18^F-labelling of trifluoromethyl-substituted molecules are of great interest for pharmaceutical drug development and the production of ^18^F-labelled PET radiotracers. In 1979, Ido reported the first synthesis of a [^18^F]CF_3_-containing compound via ^19^F/^18^F isotopic exchange (Fig. 7A) (Ido et al. 1979). The synthesis of α,α,α-[^18^F]trifluorotoluene was accomplished in yields ranging from 0.5–13% by reacting α,α,α-trifluorotoluene with [^18^F]KF and 1,4,7,10,13,16-hexaoxacyclooctadecane (18-crown-6) in benzene at 100 °C; this methodology suffers from poor yield and low reproducibility. Angelini reported in 1986 an improved radiosynthesis of α,α,α-[^18^F]trifluorotoluene upon treatment of α,α-difluoro-α-chlorotoluene and α,α,α-trichlorotoluene with [^18^F]HF and a Lewis acid (Sb_2_O_3_) (~ 50% RCY) (Fig. 7A) (Angelini et al. 1990). The method was applied to the multi-step synthesis of *N*-(α,α,α-[^18^F]trifluoro-*m*-tolyl)piperazine (serotonin agonist) in 51% RCY. More activated α-bromo-α,α-difluorotoluene-containing substrates underwent ^18^F-fluorination with [^18^F]TBAF under milder conditions than α-fluoro and α-chloro analogues to afford [^18^F]trifluoromethylated compounds in RCYs around 50% RCY as reported by Kilbourn (Fig. 7A) (Kilbourn et al. 1990). This strategy gave access to (*R*,*S*)-1-[2-[{4-[^18^F](trifluoromethyl)phenyl}methoxyethyl]-piperidine-3-carboxylic acid (GABA uptake inhibitor) in 28% RCY. Hammadi and Crouzel applied similar radiochemistry to prepare 4-chloro-[^18^F](trifluoromethyl)benzene, a precursor of [^18^F]Fluoxetine (serotonine uptake inhibitor) (9–10% RCY, A_m_ = 3.7–5.6 GBq/μmol) (Hammadi and Crouzel 1993). In 2007, Mann and Kumar reported the late-stage ^18^F-labelling of [^18^F]Celecoxib (COX-2 expression imaging) by ^18^F-fluorination of the corresponding α-bromo-α,α-difluoromethylpyrazole precursor with [^18^F]TBAF in 10% RCY and A_m_ = 4.4 GBq/μmol (Prabhakaran et al. 2007). In 2016, Gouverneur and co-workers showed that higher radiochemical yields (up to 80%) can be obtained for [^18^F]ArCF_3_ from ArCF_2_Br precursors when [^18^F]KF/Kryptofix is used in combination with AgOTf under milder conditions (rt–60 °C); Am remained low (0.03 GBq/μmol) (Verhoog et al. 2016). All these transformations required multi-step sequences for the synthesis of the precursors consisting more often of difluorination of the necessary aldehyde with DAST followed by radical bromination; also, the inherent reactivity of the α-bromo-α,α-difluorotolyl motif is incompatible with numerous functionalities. This protocol proved suitable for the synthesis of 4-[^18^F](trifluoromethyl)-1,1′-biphenyl (7% RCY) (A_m_ up to 0.25 GBq/μmol) (Fig. 7B). In 1995, methods to install [^18^F]trifluoromethyl onto alkanes were disclosed. Johnstrom reported the synthesis of the alkylating agent 2,2,2-[^18^F]trifluoroethyl triflate, which was generated in three steps from bromodifluoroacetate and [^18^F]F^−^ (Johnstrom and Stone-Elander 1995). This [^18^F]reagent granted access to (*N*-2,2,2-[^18^F]trifluoroethyl)-2-oxoquazepam in 80–85% RCY and A_m_ = 0.037 GBq/μmol. In 2011, Suehiro reported the multi-step synthesis of 1-(2-nitro-1*H*-imidazol-1-yl)-3-(2,2,2-[^18^F]trifluoroethoxy)propan-2-ol ([^18^F]TFMISO) (hypoxia marker) (A_m_ = 0.03 GBq/μmol) by *N*-alkylation of a suitable nitroimidazole precursor with [^18^F]2,2,2-trifluoroethyl tosylate, the latter synthesised by ^18^F/^19^F exchange with [^18^F]fluoride in the presence of Kryptofix (Fig. 7C) (Suehiro et al. 2011). 2,2,2-[^18^F]Trifluoroethyltosylate was also employed by Riss in *O*-alkylation for the synthesis of 1-(4-fluorobenzyl)-*N*-(1-(4-(2,2,2-[^18^F]trifluoroethoxy)phenethyl)piperidin-4-yl)-1*H*-benzo[d]imidazol-2-amine, a candidate for PET imaging of Tau pathology (Riss et al. 2011). This radiochemistry underwent various improvements (Riss and Aigbirhio 2011). In 2020, Riss and Scott implemented this protocol for the synthesis [^18^F]*N*-methyl lansoprazole ([^18^F]NML) (4.6% RCY and A_m_ = 120.1 GBq/μmol), a candidate for Alzheimer’s Tau imaging (Kramer et al. 2020). In 2019, Tredwell reported that 1,1-[^18^F]difluoroalkenes (up to 77% RCY) are accessible upon ^18^F-fluorination of fluoroalkenyl(aryl)iodonium triflates with [^18^F]KF/Kryptofix® (Fig. 7D) (Frost et al. 2019). The method enabled the automated synthesis of 4-(2,2-[^18^F]difluorovinyl)-1,1′-biphenyl in 33–47% RCY and A_m_ = 1 GBq/μmol, which was derivatised into 1-([1,1′-biphenyl]-4-yl)-2,2,2-[^18^F]trifluoroethan-1-ol (61% RCY). In 2016, [^18^F]trifluoroacetamides were obtained in up to 91% RCY from difluorobromoacetamide precursors in the presence of [^18^F]TBAF and 1,8-diazabicyclo[5.4.0]undec-7-ene (DBU) used as nucleophilic activator, a protocol reported by Szabo and Schou (Fig. 7E) (Gómez et al. 2016). The utility of this methodology was demonstrated by the synthesis of 2,2,2-[^18^F]trifluoro-1-(piperidin-1-yl)ethan-1-one in 71% RCY and A_m_ = 8.4 GBq/μmol. An alternative approach for the synthesis of [^18^F]trifluoromethylated compounds was disclosed by Marchand-Brynaert in 2001 based on oxidative ^18^F-fluorodesulfurisation of dithioates (Fig. 7F) (Josse et al. 2011). The method was applied for the multi-step synthesis of 2-(2-nitroimidazol-1-yl)-*N*-(3,3,3-[^18^F]trifluoropropyl)-acetamide ([^18^F]EF3) (hypoxia) formed in 2.9% RCY (A_m_ = 0.0026 GBq/μmol). The synthesis of [^18^F]CF_2_CF_3_-substituted radiotracers was achieved by addition of [^18^F]F_2_ to perfluoroolefin precursors. Specifically, Dolbier reported in 2001 the synthesis of [^18^F]EF5 in 17% RCY by addition of carrier-added [^18^F]F_2_ to a suitable alkene precursor (Fig. 7G). The process underwent various improvements to increase RCY and A_m_ (Dolbier et al. 2001) ^18^F-Radiolabelling of 2,2,2-[^18^F]trifluoroacetyl-containing compounds (trifluoromethyl ketones) was reported by Prakash in 2003 with the reaction of difluorinated silyl enol ethers with carrier-added [^18^F]F_2_ at low temperature (-30 °C) in acetonitrile (RCYs up to 28% RCY and A_m_ to 0.02 GBq/μmol) (Prakash et al. 2003). The synthesis of [^18^F]CF_3_-containing compounds by ^18^F-fluorodecarboxylation represented a new departure from halex-exchange reactions with [^18^F]F^−^. Building on the seminal work by Li on silver-catalysed decarboxylative fluorination of aliphatic carboxylic acids (Yin et al. 2012). Gouverneur with Luthra, Passchier and Solin developed in 2016 a methodology for the synthesis of [^18^F]trifluoromethylarenes by decarboxylative fluorination of α,α-difluoroarylacetic acids with [^18^F]Selectfluor *bis*(triflate) and AgNO_3_ (Fig. 7H) (Mizuta et al. 2013). The reaction involves a putative radical intermediate via Ag(III)-assisted single-electron transfer (SET) followed by fluorine atom transfer. 4-[^18^F](Trifluoromethyl)-1,1′-biphenyl and 4-[^18^F](difluoromethyl)-1,1′-biphenyl were obtained in ^18^% RCY (A_m_ = 0.3 GBq/μmol) and 8.6% RCY (A_m_ = 2.5 GBq/μmol), respectively.

Building on the seminal work of Burton (MacNeil and Burton 1991) and Chen (Duan 1993) the first method leading to [^18^F]trifluoromethylated (hetero)arenes applying a direct ^18^F-trifluoromethylation protocol (aryl–[^18^F]CF_3_ disconnection) was reported by Gouverneur and Passchier in 2013 (Fig. 7I) (Huiban et al. 2013). Readily available substituted aryl iodides were reacted with [^18^F]CuCF_3_, formed in situ from CF_2_ClCO_2_Me which releases a difluorocarbene trapped by [^18^F]F^−^. This operationally simple methodology requires CuI and TMEDA, and allowed the incorporation of [^18^F]CF_3_ into a broad range of (hetero)arenes bearing, ester, nitro, cyano, halide, ether, and amide functional groups, in RCYs up to 87%, and A_m_ ~ 0.1 GBq/μmol. This direct method gave immediate access to [^18^F]fluoxetine (antidepressant) and [^18^F]flutamide (prostate cancer) in 37% and 55% RCYs, respectively. Unprotected amine, alcohol, and carboxylic acid required protection due to, otherwise, competing alkylation with CF_2_ClCO_2_Me or MeI, the latter being formed as by-product of difluorocarbene formation. The method allowed direct C–H ^18^F-trifluoromethylation of indoles in 19% RCY. Following this study, various variations of this conceptually new approach appeared in the literature. For example, Vugts reported that [^18^F]CuCF_3_ can instead be generated from gaseous [^18^F]CF_3_H (Born et al. 2014). [^18^F]Trifluoromethyl (hetero)arenes were prepared from either aryl iodides (34–91% RCY) or aryl boronic acids (4–97% RCY) in the presence of CuBr and Et_3_N·3HF. ^18^F-Trifluoromethylation of boronic acid precursors was conducted under milder conditions (rt) compared to aryl iodide precursors (130 °C) and required air purging of the reaction mixture. Unexpectedly, the presence of Et_3_N·3HF was not detrimental to molar activity (A_m_ up to 32 GBq/μmol). This methodology afforded 1-[^18^F]trifluoromethyl-4-nitrobenzene, *N*-(*tert*-butoxycarbonyl)-4-[^18^F](trifluoromethyl)-L-phenylalanine methyl ester and 3-deoxy-3-[^18^F](trifluoromethyl)estrone in up to 91%, 89% and 73% RCYs, respectively (Fig. 7I). In the same year, Riss developed another variation of this Cu(I)-mediated ^18^F-trifluoromethylation using [^18^F]CF_3_H, KHCO_3_, CuBr and *N*,*N*-diisopropyl-*N*-ethylamine (DIPEA) (Fig. 7I) (Rühl et al. 2014). Finally, Pannecoucke reported the use of *S*-(difluoromethyl)diarylsulfonium salts for the synthesis of [^18^F]CF_3_H with [^18^F]KF or [^18^F]TBAF, subsequently converted into [^18^F]CuCF_3_ species in the presence of K[Cu(O*t*Bu)_2_] (Fig. 7I) (Ivashkin et al. 2014). Aryl boronic acid and aryl iodide precursors were reacted with stock solutions of [^18^F]CuCF_3_ to afford [^18^F]CF_3_-(hetero)arenes in up to 88% RCY. ^18^F-radiolabelling reactions were conducted at high temperature (100 °C) under inert atmosphere (N_2_) in the presence of B(OMe)_3_, which is required to neutralise the excess of *t*BuOK necessary for [^18^F]CuCF_3_ formation. This method afforded [^18^F]*N*-Boc-Fluoxetine (85% RCY, A_m_ not reported) and [^18^F](trifluoromethyl)nitrobenzene (78% RCY, A_m_ = 0.1 GBq/μmol).

In 2020, Gouverneur and co-workers disclosed a methodology to access [^18^F]trifluoroethyl ethers (Fig. 7J) (Pees et al. 2021). The protocol employs [^18^F]trifluoroethanol, synthesised in two steps from bromodifluoroacetate precursors and [^18^F]TBAF; this ^18^F-reagent is subsequently cross-coupled with (hetero)aryl bromides in the presence of *t*BuBrettPhosPdG3. This strategy afforded 4-(2,2,2-[^18^F]trifluoroethoxy)phenyl)morpholine and 2-(2,2,2-[^18^F]trifluoroethoxy)naphthalene in 27% and 15% RCY, respectively. Building on Groves seminal studies (Liu et al. 2018; Huang et al. 2014, 2015). Carroll reported in 2015 the oxidative benzylic C–H ^18^F-fluorination of difluorinated benzylic precursors (Fig. 7K) (Carroll et al. 2015). The protocol employed Mn complexes, [^18^F]F^−^, PhIO, ^18^-crown-6 and AgOTf to afford ^18^F-labelled derivatives in 3–72% RCY. For example, 1-bromo-4-[^18^F](trifluoromethyl)benzene was synthesised in 44% RCY and A_m_ = 4 GBq/μmol. In 2021, Vugts and co-workers disclosed a protocol for the radiosynthesis of the Ruppert-Prakash reagent [^18^F]Me_3_SiCF_3_ from [^18^F]CF_3_H (A_m_ = up to 13 GBq/μmol) (Fig. 7L) (Pees et al. 2020, 2021; Yang et al. 2019). This methodology employs a combination of [^18^F]CHF_3_, trimethylsilyl chloride (Me_3_SiCl), potassium hexamethyldisilazide (KHMDS) and tetrabutylammonium fluoride (TBAF) or tetrabutylammonium difluorotriphenylsilicate (TBAT) that react at low temperature (− 78 °C) to afford [^18^F]Me_3_SiCF_3_. After distillation, this ^18^F-labelled reagent is reacted with acetophenone, benzaldehyde and benzophenone to afford the addition [^18^F]trifluoromethylated products in up to 96% RCY. Toste reported in 2017 a conceptually novel approach to ^18^F-labelled alkyl-CF_3_ featuring bis(trifluoromethyl) gold complexes (Fig. 7M) (Levin et al. 2017). The proposed mechanism involves fluoride abstraction from a CF_3_ moiety from the initial [AuIPr(CF_3_)_2_R] complex (IPr = 1,3-bis(2,6-diisopropylphenyl)-1,3-dihydro-2H-imidazol-2-ylidene, R = alkyl) by B(C_6_F_5_)_3_, resulting in the formation of a difluorocarbenoid intermediate. This is subsequently transformed into an isolable Au(III)–CF_2_R (R = alkyl) complex capable of furnishing [^18^F]alkylCF_3_ via reductive elimination in the presence of [^18^F]fluoride-Kryptofix®. This methodology afforded 1-(4-(4,4,4-[^18^F]trifluorobutyl)phenyl)ethan-1-one and 5,5,5-[^18^F]trifluoropentyl-2-naphthoate in 21% and 27% RCYs, respectively. The radiosynthesis of [^18^F]BAY 59-3074 (cannabinoid receptor partial agonist) was accomplished in 6% RCY and A_m_ = 0.3 GBq/μmol.

#### Radiosynthesis of [^18^F]difluoromethyl-containing molecules

**Fig. 8:**
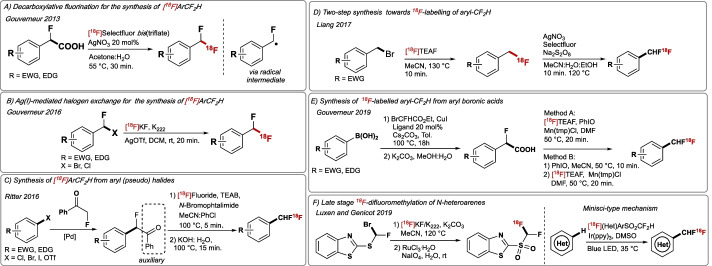
**A** Radiosynthesis of [^18^F]difluoromethylarenes via oxidative decarboxylation with Selectfluor *bis*(triflate). **B** Radiosynthesis of [^18^F]difluoromethylarenes via halogen exchange. **C** Two-step approach from aryl (pseudo) halides. **D** Two-step approach from benzyl bromides. **E** Two-step approach from aryl boronic acids. **F** New disconnection: late-stage radical ^18^F-difluoromethylation with Hu-type [^18^F]reagent. EWG = electron-withdrawing group. EDG = electron-donating group

The highly polarised C–H bond of CF_2_H makes this motif a competent hydrogen bond donor, a characteristic unique amongst polyfluorinated motifs. The suitability of CF_2_H as a bio-isostere for alcohol, thiol, or amine, has resulted in its incorporation in drugs, herbicides, fungicides, and agrochemicals (Zafrani et al. 2019; Sap et al. 2021). Consequently, methods for accessing [^18^F]CF_2_H-substituted molecules for PET imaging purpose are in demand. In 2013, Gouverneur reported a Ag-mediated ^18^F-fluorodecarboxylation towards [^18^F]difluoromethylated arenes ([^18^F]ArCF_2_H) using [^18^F]Selectfluor as ^18^F-source (Huiban et al. 2013). This protocol led to 1-(*tert*-butyl)-4-[^18^F](difluoromethyl)benzene in 10% RCY and A_m_ up to 0.03 GBq/μmol (Fig. 8A). To develop a method using [^18^F]fluoride, halogen exchange has proven to be a viable approach for the preparation of [^18^F]difluoromethyl arenes in the presence of AgOTf (Gouverneur 2016) (Fig. 8B) (Verhoog et al. 2016) In the same year, Ritter disclosed a methodology towards the synthesis of [^18^F]difluoromethylarenes from aryl (pseudo)halides precursors, [^18^F]fluoride and the electrophilic bromination reagent (*N*-bromophtalimide) (Fig. 8C) (Shi et al. 2016). The method requires Pd-catalysed cross-coupling chemistry for the synthesis of the starting materials. [^18^F]CHF_2_-Fenofibrate, [^18^F]CHF_2_-Boc-fluoxetine and 6-[^18^F]difluoromethylquinoline were prepared in 39%, 17% and 43% RCY, respectively. Liang reported one year later a two-step synthesis of [^18^F]ArCHF_2_ from variously functionalised benzylic (pseudo)halides (Fig. 8D) (Yuan et al. 2017). This method proceeds via S_N_2 with [^18^F]F^−^ followed by oxidative C–H radical fluorination of the ^18^F-labelled products with Selectfluor and Na_2_S_2_O_8_ to afford [^18^F]ArCHF_2_ in 10–45% RCY; as expected, electron-rich substrates were not amenable to this protocol. This strategy afforded 4-[^18^F](difluoromethyl)-1,1’-biphenyl in 23% RCY and A_m_ = 22 GBq/μmol. Building on previously disclosed Ag(I)-mediated ^18^F-fluorodecarboxylation with [^18^F]Selectfluor *bis*(triflate), Gouverneur developed in 2019 a nucleophilic approach towards the synthesis of [^18^F]difluoromethylarenes employing aryl boronic acids, ethyl bromofluoroacetate and [^18^F]tetraethylammonium fluoride ([^18^F]TEAF) (Fig. 8E) (Sap et al. 2019). The reaction proceeds via Cu-catalysed cross-coupling of aryl boronic acids with ethyl bromofluoroacetate, followed by in situ hydrolysis affording α-fluoroarylacetic acids, which undergo Mn-mediated [^18^F]fluorodecarboxylation in the presence of Mn(temp)Cl, iodosylbenzene and [^18^F]F^−^ to furnish [^18^F]ArCHF_2_ in up to 32% RCY. 1-[^18^F](Difluoromethyl)-4-phenoxybenzene was obtained in 12% RCY (d.c) and A_m_ = 3 GBq/μmol. This protocol displays a wide functional group tolerance including alkyl, alkoxy, bromo, iodo and aldehyde, and was also suitable for the synthesis of an [^18^F]OCF_2_H derivative of Estrone in 21% RCY. In 2019, Genicot and Luxen reported a strategy featuring C–[^18^F]CF_2_H bond formation upon radical ^18^F-difluoromethylation of heteroarenes (Fig. 8F) (Trump et al. 2019). The method employs the new ^18^F-labelled reagent [^18^F]((2-difluoromethyl)sulfonyl)benzo[*d*]thiazole (Hu reagent) (Rong et al. 2016), which was synthesised in two steps from 2-((bromofluoromethyl)thio)benzo[*d*]thiazole and [^18^F]F^−^/K_222_, followed by oxidation (NaIO_4_, RuCl_3_) (13.4% RCY, A_m_ = 81 GBq/μmol), decay corrected (dc); this Am value is the highest reported for the labelling of CF_2_H. This reagent activated in the presence of Ir(ppy)_3_ and light (blue LED) enabled radical ^18^F-difluoromethylation of *N*-heteroarenes in up to 75% RCY; the more challenging arenes have not been subjected ^18^F-difluoromethylation applying this methodology. The method led to [^18^F]acyclovir (antiherpetic) in 70% RCY and a molar activity of 35 GBq/μmol. Automation was demonstrated on the ‘AllinOne’ TRASIS platform (Trump et al. 2020).

#### Radiosynthesis of [^18^F]SCF_3_, SCHF_2_, OCF_3_ and OCHF_2_-containing molecules

**Fig. 9:**
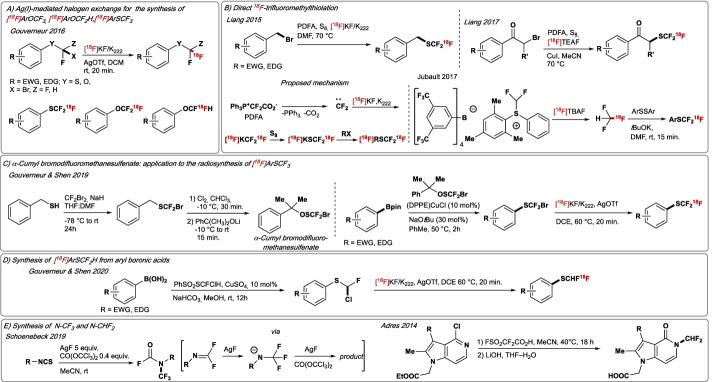
**A **Radiosynthesis of [^18^F]ArOCF_3_, [^18^F]ArOCF_2_H, and [^18^F]ArSCF_3_ by Ag-mediated halogen exchange with [^18^F]fluoride. **B **[^18^F]trifluoromethylthiolation with difluorocarbene precursors and [^18^F]fluoride. **C** Radiosynthesis of [^18^F]ArSCF_3_ from aryl boronic esters. **D** Radiosynthesis of [^18^F]ArSCHF_2_ from aryl boronic acids. **E** Synthesis of N-CF_3_ and N-CF_2_H. EWG = electron-withdrawing group, EDG = electron-donating group

Medicinal chemists have employed di-and trifluoromethyl ether, as well as thioether substitution to tune conformation on demand, or modulate physicochemical parameters such as lipophilicity (Landelle et al. 2014). Pharmaceutical drugs that feature these substitutions are the CF_3_O-containing 2-amino benzothiazole riluzole, for the treatment of amyotropic lateral sclerosis (Bellingham 2011) or the CF_3_S-substituted 2-phenylethylamine tiflorex (Silverstone et al. 1979) which possesses anorectic activity. The availability of these motifs in their ^18^F-labelled form is therefore of importance and timely. Gouverneur disclosed in 2015 a ^18^F-methodology towards [^18^F]ArOCF_3_, [^18^F]ArOCF_2_H and [^18^F]ArSCF_3_ via Ag(I)-mediated halogen exchange (Khotavivattana et al. 2015). The reaction proceeds under mild conditions (rt—60 °C) and requires [^18^F]KF/Kryptofix® and AgOTf. The method afforded ^18^F-labelled products in up to 81% RCY and A_m_ ~ 0.1 GBq/μmol (Fig. 9A). In the same year, Xiao and Liang developed a metal-free difluorocarbene-derived protocol for ^18^F-trifluoromethylthiolation of aliphatic, benzylic halides (Fig. 9B) (Zheng et al. 2015). The reaction was performed with Ph_3_P^+^CF_2_CO_2_^−^ (difluoromethylene phosphobetaine, PDFA) as difluorocarbene source, which reacts with [^18^F]KF/Kryptofix, elemental sulfur (S_8_) and benzyl bromides to afford the corresponding [^18^F]trifluoromethylthiolated products (37–53% RCY). In 2017, this group extended this methodology towards the synthesis of α-SCF_3_ carbonyl derivatives (Zheng et al. 2017). Optimised conditions employed PDFA, S_8_, [^18^F]TEAF and CuI. The presence of copper facilitated the formation of a [^18^F]CuSCF_3_ species, which underwent net substitution to furnish the [^18^F]SCF_3_ products. Jubault and Labar disclosed in 2017 a route to [^18^F]SCF_3_-substituted arene derivatives by reacting [^18^F]CHF_3_, generated from the bench-stable (difluoromethyl)-(mesityl)(phenyl) sulfonium salt, with disulfides in the presence of *t*BuOK (Fig. 9B) (Carbonnel et al. 2017). ^18^F-Labelled trifluoromethylated compounds were obtained in up to 75% RCY and A_m_ ~ 0.38 GBq/μmol. In 2019, Shen and Gouverneur reported the synthesis of α-cumyl bromodifluoromethanesulfenate, a novel reagent enabling the direct Cu-mediated conversion of (hetero)aryl boronic esters into bromodifluoromethylthiolated (hetero)arenes (Fig. 9C) (Wu et al. 2019). These readily assembled substrates reacted with [^18^F]KF/Kryptofix in the presence of AgOTf to furnish [^18^F]SCF_3_-arenes in up to 86% RCY and A_m_ ~ 0.1 GBq/μmol. In 2020, Shen and Gouverneur also reported the ^18^F-labelling of [^18^F]difluoromethylthio-containing compounds ([^18^F]SCHF_2_), a motif found in several drug-like molecules such as β-lactamase resistant oxcephalosporin, flomoxef sodium (antibiotic) or pyriprole (pesticide) (Fig. 9D) (Zhao et al. 2020). The protocol features a copper-catalyzed chlorofluoromethylthiolation of (hetero)aryl boronic acids with the novel reagent PhSO_2_SCFClH; the resulting cross-coupled products subsequently reacted with [^18^F]KF/Kryptofix® to afford a range of [^18^F]ArSCHF_2_ products in up to 81% RCY. For example, [^18^F]SCHF_2_ analogues of clofibrate and estrone were synthesised in 73% and 15% RCY, respectively. The molar activity was found to be 0.12 GBq/μmol.

Motifs that have been less explored are N-CF_3_(Milcent and Crousse 2018) and N-CF_2_H (Gaba and Mohan 2016) but with the continuous development of new reagents and methods, the prevalence of these groups may increase for example in *N*-heteroaromatic scaffolds such as imidazoles and benzimidazoles that are commonly encountered in medicinal chemistry. We noted the report of Schoenebeck and co-workers that offers a method to access *N*-trifluoromethylcarbonyl derivatives from a bench-stable carbamoyl fluoride *N*-trifluoromethylated reagent (Scattolin et al. 2019) (Fig. 9E). Methods for the attachment of a CF_2_H group into a N–H bond have also been described, offering new opportunities for medicinal and agricultural chemistry. For example, Andrés demonstrated that the receptor residence times in a family of pyridone-containing CRTh2 antagonists can be modulated by varying the substituent at the pyridine nitrogen (Andre´s et al. 2014). N-Difluoromethyl 2-pyridones had a significantly higher dissociation half-life compared with N-unsubstituted or *N*-methylated pyridines (Fig. 9E). To date, no methods have been reported to ^18^F-label these motifs. Such new technologies would bring new information on metabolism, receptor occupancy, and beyond.

### ^18^F-Labelling of biomolecules via ^18^F–C bond construction

**Fig. 10:**
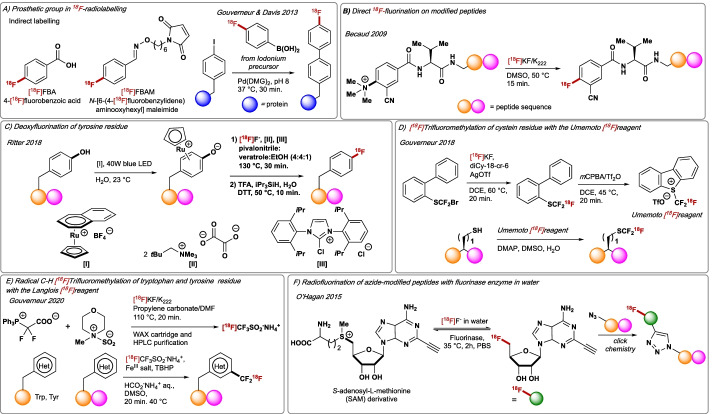
**A** Use of prosthetic groups for indirect radiofluorination of peptides. **B** Direct ^18^F-incorporation on modified peptides. **C** Ru-mediated deoxyfluorination of peptidic tyrosine. **D** [^18^F]Umemoto reagent for radiofluorination of peptidic cysteine. **E** C–H ^18^F-Trifluoromethylation of peptidic tryptophan and tyrosine with the [^18^F]Langlois reagent. **F** Radiofluorination with fluorinase enzyme. DMG = dimethylguanidine. TFA = trifluoroacetic acid. DTT = DL-dithiothreitol

Notwithstanding their importance, the development of _18_F-labelling methods for biomolecules that rely on phosphorus-, aluminium-, boron-, sulfur- and silicon-fluorine bond formation from ^18^F-fluoride will not be discussed in this account as this topic has been reviewed elsewhere (Bernard-Gauthier et al. 2018). Direct ^18^F-labelling of biomolecules including peptides or proteins in aqueous medium via C–^18^F bond construction is challenging due to ^18^F-fluoride solvation and the harsh conditions typically required for such reaction e.g. high temperatures (> 100 °C). For these reasons, prosthetic groups are widely used enabling indirect ^18^F-labelling of biomolecules that are not amenable to last step ^18^F-incorporation (Schirrmacher et al. 2017). This approach requires the installation onto the biomolecule of a prosthetic group, the radiosynthesis of the ^18^F-radiolabelled motif that will react with the prosthetic, and a final coupling step. Attachment of the prosthetic group to the biomolecule exploits the inherent reactivity of natural amino acids, or unnatural amino acids that must be introduced applying chemical or biochemical methods. More often, lysine residues are coupled with activated carboxylic acid groups or via reductive amination, and cysteine thiols are reacted with maleimides under mild conditions. Selected examples of prosthetic groups include *N*-succinimidyl 4-[^18^F]fluorobenzoate ([^18^F]SFB) (Vaidyanathan and Zalutsky 1992) 4-[^18^F]fluorobenzoic acid ([^18^F]FBA) (Marik and Sutcliffe 2007), N-[6-(4-[^18^F]fluorobenzylidene)aminooxyhexyl] maleimide ([^18^F]FBAM) (Li et al. 2008) and *N*-[2-(4-[^18^F]fluorobenzamido)ethyl]maleimide ([^18^F]FBEM) (Kiesewetter et al. 2011) (Fig. 10A). 4-[^18^F]Fluorobenzaldehyde has been used in multi-component reactions e.g. Igu, Biginelli, Groebke, and Passerini to construct peptidic-like amide linkages as well as heterocycles (Gouverneur, 2011) (Li et al. 2011). More recently, Davis and Gouverneur have reported the first Pd-mediated attachment of an ^18^F-labelled aryl boronic acid prosthetic group onto proteins, a process enabled with an effective Pd ligand for aqueous Suzuki–Miyaura coupling at low substrate concentrations (0.10–0.20 mM). This discovery further expands the scope of aqueous Pd catalysis to radiobiology (Fig. 10A). The use of prosthetic groups for the direct ^18^F-labelling of peptides was firstly reported by Becaud in 2009 (Fig. 10B) (Becaud et al. 2009). Direct ^18^F-fluorination of model trimethylammonium-substituted modified peptides with [^18^F]KF-Kryptofix enabled the synthesis of ^18^F-labelled tetrapeptides and analogues of bombesin peptides under relatively mild conditions. 3-Cyano-4-[^18^F]fluorobenzoyl-Ava-Gln-Trp-Ala-Val-Gly-HisFA01010-Leu-NH_2_ was formed in 21% RCY and A_m_ = 73 GBq/μmol. This methodology has demonstrated usefulness towards direct ^18^F-fluorination of peptides containing histidine, tryptophan, lysine, and arginine residues without the need of protecting groups. Despite the successful use of prosthetic groups for PET applications, methodologies amenable to late stage or last step ^18^F-functionalisation of biomolecules are very much desired. If strategically positioned, fluorine substitution should cause minimal impact on the native structure and function of large biomolecules. Several groups have contributed to addressing such challenges by deploying a range of strategies. Britton and co-workers reported an elegant site-selective photocatalytic C–H ^18^F-fluorination of leucine residue within complex peptide using [^18^F]F_2_-derived [^18^F]NFSI (details illustrated in Fig. 1C) (Nodwell et al. 2017; Yuan et al. 2018) and Ritter disclosed a Ru-mediated ^18^F-deoxyfluorination at tyrosine which proved suitable for the synthesis of an analogue of neuromedin B (bombesin-related peptide) containing 10 amino acids (Fig. 10C) (Rickmeier and Ritter 2018). In 2018, Gouverneur reported the ^18^F-labelling of 5-[^18^F](trifluoromethyl)dibenzothiophenium trifluoromethanesulfonate, an ^18^F-isotopologue of the Umemoto reagent which was synthesised by halogen exchange with [^18^F]fluoride to afford [1,1′-biphenyl]-2-yl(difluoro(fluoro-^18^*F*)methyl)sulfane that underwent a subsequent oxidative cyclisation (Fig. 10D) (Verhoog et al. 2018). The [^18^F]Umemoto reagent allowed access to [^18^F]trifluoromethylated-cysteine residues in native unprotected peptides with high chemoselectivity as exemplified by the synthesis of arginylglycylaspartic acid[Cys(CF_2_^18^F)] (RGD) (19% RCY). This radiotracer enabled in vivo PET imaging studies that demonstrated that this new cysteine-derived [^18^F]SCF_3_ motif does not undergo in vivo β-elimination, a process that would lead to [^18^F]fluoride release and increased bone uptake. Inspired by studies carried out in the groups of Davis (Gao et al. 2013) and of Krska (Ichiishi et al. 2018). Gouverneur reported in 2020 the radical C–H ^18^F-trifluoromethylation of unprotected peptides at tryptophan and tyrosine residues with the novel ^18^F-reagent ammonium [^18^F]trifluoromethanesulfinate ([^18^F]CF_3_SO_2_NH_4_) ([^18^F]Langlois reagent); this [^18^F]reagent was accessible in a single step from [^18^F]KF-Kryptofix, the difluorocarbene precursor 2,2-difluoro-2-(triphenylphosphonio)acetate (PDFA) and the *N*-methylmorpholine·SO_2_ (NMM·SO_2_) complex (Fig. 10E) (Kee et al. 2020). The method which entailed the treatment of the peptide with [^18^F]CF_3_SO_2_NH_4_ in the presence of *tert*-butyl hydroperoxide (TBHP) and Fe(NO_3_)_3_·9H_2_O/FeCl_3_ in DMSO/aqueous ammonium formate (25 mM), displayed chemoselectivity for tryptophan and tyrosine residues, and granted access to various [^18^F]peptides including insulin[Tyr(CF_2_^18^F)] (34% RCY). The automated synthesis of octreotide[Trp(CF_2_^18^F)], an octapeptide that mimics natural somatostatin (growth hormone secretion inhibitor), was carried out in 29% RCY and A_m_ = 0.28 GBq/μmol. The discovery of the naturally occurring fluorinase enzyme, originally isolated from *Streptomyces cattleya*, represented a major breakthrough in biocatalytic fluorination as reported by O’Hagan in 2002 (Fig. 10F) (O′Hagan et al. 2002). This enzyme catalyses the reaction between S-adenosyl-L-methionine (AdoMet) and F^−^ in aqueous media to afford 5’-fluoro-5’-deoxyadenosine (FDA) and L-methionine (L-Met) following a S_N_2 nucleophilic substitution pathway. This methodology has proven suitable for enzymatic ^18^F-fluorination with overexpressed recombinant fluorinase as exemplified by the synthesis of [^18^F]FDA in RCYs up to 95% (Thompson et al. 2015). In 2019, O’Hagan discovered that halogenated-5′-deoxy adenosine, 5’-chloro-5’-deoxyadenosine (5′-ClDA), 5’-bromo-5’-deoxyadenosine (5′-BrDA), and to a lesser extent 5′-IDA, are competent substrates for transhalogenation reaction with fluorinase enzyme in presence of S-adenosyl-L-methionine (AdoMet) or Se-adenosylselenomethionine (AdoSeMet) (Lowe et al. 2019). Enzymatic fluorinase-mediated radiofluorination was also applied for the last step ^18^F-fluorination of RGD peptides, A_2_A adenosine receptor agonists, as well as tetrazine and biotin motifs (Thompson et al. 2015). For example, cancer-relevant targeting peptides [^18^F]FDEA-TEG-RGD and [^18^F]FDA-PEG-GUL were ^18^F-radiolabelled with aqueous [^18^F]F^−^ in 12% and 3.4% RCY, respectively, under physiological conditions (pH = 7.8, 37 °C) in scales sufficient for preclinical in vivo PET studies.

## Conclusions

Significant progress in ^18^F-radiochemistry has been made in the last two decades with approaches that have improved or departed from conventional nucleophilic (aromatic) substitution. Lessons have been learned from the spectacular progresses made in late stage ^19^F-fluorination, that led to novel ^18^F-labelled reagents and the inclusion of numerous activation manifolds to facilitate ^18^F-incorporation. Nowadays, transition metals, photoredox catalysts and organocatalysts are routinely considered in the development of novel ^18^F-fluorination methodologies. Although the gap between ^19^F and ^18^F chemistry has narrowed significantly, long-standing challenges remain to warrant unlimited access ^18^F-labelled fluoro(hetero)arenes. Numerous highly effective late stage ^19^F-fluorination methodologies make use of electrophilic fluorinating *N*–F reagents, and the successful labelling of some of these reagents including [^18^F]NFSI and [^18^F]Selectfluor have demonstrated their huge potential for radiotracers development. However, this field will require improved methods for labelling these *N*–F reagents using cyclotron-produced [^18^F]fluoride, and circumventing the handling of molecular fluorine; such methods must also improve on molar activity to enable a broader range of imaging studies. Methodologies that allow the installation of ^18^F-perfluorinated motifs (–CF_3_, –CF_2_H, –SCF_3_, –OCF_3_, –OCF_2_H, –CF_2_CF_3_, or –SF_5_) should be further investigated in the upcoming years in response to their increasing prevalence in medicinal chemistry and drug discovery. Most methods have applied halogen exchange with [^18^F]fluoride, but new pathways that assemble these perfluorinated motifs in their ^18^F-labelled form from [^18^F]fluoride have materialised; these include multi-component reactions generating [^18^F]CF_3_^−^ or [^18^F]CF_3_S^−^ in situ for direct cross-couplings or substitution. The key challenge to overcome is molar activity that remains low compared to what is achievable for ^18^F-monofluorinated motifs. ^18^F-Reagent development for ^18^F-tri- and ^18^F-difluoromethylation has made steady progress with [^18^F]CuCF_3_, [^18^F]CF_3_H, [^18^F]Umemoto reagent, [^18^F]Langlois reagent, [^18^F]Hu reagent, and [^18^F]Ruppert-Prakash reagent all available. More generally, methods enabling site-selective C–H functionalisation of ^18^F-motifs are underdeveloped; this state of play will likely progress when further advances in late-stage chemoselective C–H ^19^F-functionalisation are made. For radiochemistry, such C–H functionalisation must consider whether separation of the ^18^F-labelled molecule from starting material is rapidly achievable. An area that has recently received increased attention is the ^18^F-labelling of biomolecules, mainly peptides and proteins. Traditional methods feature indirect labelling strategies based on prosthetic chemistry, but direct ^18^F-fluorination/perfluoroalkylation methods have appeared although they are still very limited. A striking example is the fluorinase-mediated nucleophilic ^18^F-fluorination of modified peptides with [^18^F]fluoride that enables C–^18^F bond construction under very mild reaction conditions. Further development should consider control over site selectivity. In future years, we anticipate that technologies such as flow chemistry combined with the appearance of innovative methods for ^18^F-incorporation will continue to accelerate progress in PET radiotracer development. Impact for any novel method will require demonstrated applicability for (pre)clinical usage. This is not a trivial transition considering the additional challenges that one must consider including automation, and radiolysis if a protocol is to be used for multi-dose production of complex radiotracers. Finally, there is no doubt that the need for superior ^18^F-labelling methods has inspired new developments in late-stage ^19^F-fluorination. Our own research program has focused on the issue of late-stage nucleophilic fluorination with metal alkali fluoride including KF, thereby providing new tools to control fluoride reactivity (Pupo et al. 2018, 2019; Roagna et al. 2020; Ibba et al. 2020). It is this multi-way communication between chemists, radiochemists, and clinicians that has enriched fluorine chemistry enormously in recent years for the benefit of all scientists interested in the synthesis and applications of high value fluorine-containing (radio)pharmaceuticals.

## Data Availability

Not applicable.

## Abbreviations

[^18^F]AcOF: Acetyl [^18^F]hypofluorite
[^18^F]DAA1106: *N*-(2,5-dimethoxybenzyl)-*N*-(5-[^18^F]fluoro-2-phenoxyphenyl)acetamide
[^18^F]EF3: 2-(2-nitroimidazol-1-yl)-*N*-(3,3,3-[^18^F]trifluoropropyl)-acetamide
[^18^F]EF5: 2-(2-nitro-1*H*-imidazol-1-yl)-*N*-(2,2,3,3,3-[^18^F]pentafluoropropyl)-acetamide
[^18^F]FBA: 4-[^18^F]fluorobenzoic acid
[^18^F]FBAM: *N*-[6-(4-[^18^F]fluorobenzylidene)aminooxyhexyl] maleimide
[^18^F]FBEM: *N*-[2-(4-[^18^F]fluorobenzamido)ethyl]maleimide
[^18^F]FClO_3_: Perchloryl [^18^F]fluoride
[^18^F]FDG: [^18^F]fluorodeoxyglucose
[^18^F]FLT: 3′-deoxy-3′-[^18^F]fluorothymidine
[^18^F]FMISO: 1-(2-nitro-imidazolyl)-3-[^18^F]fluoro-2-propanol
[^18^F]FMT: 6-[^18^F]fluoro-L-*m*-tyrosine
[^18^F]FMTEB: 3-[^18^F]fluoro-5-((2-methylthiazol-4-yl)ethynyl)benzonitrile
[^18^F]FPEB: 3-[^18^F]fluoro-5-(pyridin-2-ylethynyl)benzonitrile
[^18^F]FU: 5-[^18^F]fluorouracil
[^18^F]MFBG: *m*-[^18^F]fluorobenzylguanidine
[^18^F]NFP: [^18^F]*N*-fluoropyridinium
[^18^F]NFSI: [^18^F]*N*-fluorobenzenesulfonimide
[^18^F]NML: [^18^F]*N*-methyl lansoprazole
[^18^F]Selectfluor *bis*(triflate): 1-chloromethyl-4-[^18^F]fluoro-1,4-diazoniabicyclo-[222]octane *bis*(triflate)
[^18^F]SFB: *N*-succinimidyl 4-[^18^F]fluorobenzoate
[^18^F]TBAF: *n*-tetrabutylammonium [^18^F]fluoride
[^18^F]TEAF: Tetraethylammonium [^18^F]fluoride
[^18^F]TFMISO: 1-(2-nitro-1*H*-imidazol-1-yl)-3-(2,2,2-[^18^F]trifluoroethoxy)propan-2-ol
[bmim][OTf]: 1-ethyl-3-methylimidazolium triflate
[emim][BF_4_]: 1-butyl-3-methylimidazolium tetrafluoroborate
18-crown-6: 1,4,7,10,13,16-hexaoxacyclooctadecane
6-[^18^F]FDA: 6-[^18^F]fluorodopamine
6-[^18^F]FDOPA: 3,4-dihydroxy-6-[^18^F]fluoro-L-phenylalanine
AEX: Anion-exchange resin
AlkylFluor: 1,3-bis(2,6-diisopropylphenyl)-2-fluoroimidazolium tetrafluoroborate
A_m_: Molar activity
AY: Activity yield
cGMP: Current good manufacturing practice
CH_3_CN: Acetonitrile
CRA-S_N_Ar: Cation-radical-accelerated S_N_Ar nucleophilic aromatic substitution
CS_N_Ar: Concerted nucleophilic aromatic substitution
DAST: Diethylaminosulfur trifluoride
DBTO: Dibenzothiophene *S*-oxide
Deoxo-Fluor: bis(2-methoxyethyl)aminosulfur trifluoride
DIPEA: *N*,*N*-diisopropyl-*N*-ethylamine
DMAP: *N*,*N*-dimethylpyridin-4-amine
DMAPH^+^TfO^−^: Dimethylaminopyridinium trifluoromethanesulfonate
DMF: Dimethylformamide
DMSO: Dimethylsulfoxide
DOE: Design of experiments
EDG: Electron-donating group
EWG: Electron-withdrawing group
FG: Functional group
Ils: Ionic liquids
IPr: 1,3-bis(2,6-diisopropylphenyl)-1,3-dihydro-2H-imidazol-2-ylidene
K_222_: Kryptofix®
KHMDS: Potassium hexamethyldisilazide
LG: Leaving group
Me_3_SiCl: Trimethylsilyl chloride
N5-[^18^F]FAO: N5-[^18^F]fluoroacetylornithine
NCS: N-Chlorosuccinimide
Ndc: Non-decay-corrected
NHC: *N*-heterocyclic carbene
NMe_4_F: Tetramethylammonium fluoride
NMM·SO_2_: *N*-methylmorpholine·SO_2_
OTf: Triflate
OTs: Tosylate
PARP: Poly(ADP-ribose) polymerase
PC: Photocatalyst
PDFA: 2,2-difluoro-2-(triphenylphosphonio)acetate
PET: Positron emission tomography
PhenoFluor: 1,3-bis(2,6-diisopropylphenyl)-2,2-difluoro-2,3-dihydro-1H-imidazole
PhthH: *N*-Hydroxyphthalimide
PIDA: Phenyliodine diacetate
PTA: Phase transfer agent
PyFluor: Pyridine-2-sulfonyl fluoride
QMA: Quaternary methylammonium
RCY: Radiochemical yield
SET: Single electron transfer
S_N_2: Bimolecular nucleophilic aliphatic substitution
S_N_Ar: Nucleophilic aromatic substitution
TBAB: Tetrabutylammonium bicarbonate
TBAT: Tetrabutylammonium difluorotriphenylsilicate
TBHP: *tert*-butyl hydroperoxide
TBPA: *tert*-butyl peroxyacetate
TFAA: Trifluoroacetic anhydride
TfOH: Triflic acid
