# Correction to: Closing the gap between ^19^F and ^18^F chemistry

**DOI:** 10.1186/s41181-021-00152-x

**Published:** 2022-01-10

**Authors:** Javier Ajenjo, Gianluca Destro, Bart Cornelissen, Véronique Gouverneur

**Affiliations:** 1 Medical Research Council Oxford Institute for Radiation Oncology, University of Oxford, Oxford, OX3 7DQ UK; 2 Chemistry Research Laboratory, Department of Chemistry, University of Oxford, Oxford, OX1 3TA UK

## Correction to: EJNMMI radiopharm. chem. (2021) 6:33 10.1186/s41181-021-00143-y

Following the publication of the original article (Ajenjo et al. 2021), the authors provided clarifications, additional information, and changes in the text and reference list.

The changes have been highlighted and are shown in Additional file 1.

The original article (Ajenjo et al. 2021) has been corrected.

## Supplementary Information

**Additional file 1**.
