# Supplementary material for: Correction to: Closing the gap between 19F and 18F chemistry
Source: EJNMMI Radiopharm Chem. 2022 Jan 10;7:1. doi: 10.1186/s41181-021-00152-x (PMC8748566; doi:10.1186/s41181-021-00152-x)
Supplement: Supplementary file 1 — Additional file 1. [file 41181_2021_152_MOESM1_ESM.docx]

Following publication of the original article [1], the authors identified an error in the article body.

“Should be changed with” has become “🡪 “

In blue are highlighted general mistakes

In green are highlighted missing reference from the original manuscript

I**NTRODUCTION**

“late-stage (Hiller et al. 2018) F-fluorination” 🡪 “late-stage ^19^F-fluorination”

“(Jacobson et al. 2015; Eskola et al.2012) F-Radiopharmaceuticals” 🡪 “(Jacobson et al. 2015) ^18^F-Radiopharmaceuticals”

“between (Hiller et al. 2018). F-chemistry” 🡪 “between ^19^F-chemistry”

“late-stage (Hiller et al. 2018). F-fluorination” 🡪 “late-stage ^19^F-fluorination”

**18F‑Fluorination with [18F]F2 and [18F]F2‑derived reagents**

“nuclear reactions (Adam et al. 1986) Ne(d,α)18F” 🡪 “nuclear reactions ^20^Ne(d,α)^18^F”

“(6-[18F]FDOPA) (Eskola et al. 2012)” 🡪 “(6-[^18^F]FDOPA) Forsback et al. 2008)”

**From now on the references are shifted by one (see the examples below)**

“([18F]EF5) (Hiller et al. 2018)” 🡪 “([^18^F]EF5) (Eskola et al. 2012)”

“([18F]FClO3) (Adam et al. 1986)” 🡪 “([^18^F]FClO_3_) (Hiller et al. 2008)”

“([18F]AcOF) (Chirakalet al. 1984)” 🡪 ([^18^F]AcOF) (Adam et al. 1986)”

“[18F]XeF2, (Lu and Pike 2010)” 🡪 [^18^F]XeF_2_ (Chirakal et al. 1984)”

“with [^18^F]F^−^ (Firnau et al. 1980)” 🡪 “with [^18^F]F^−^(Lu et al. 2010)”

“with [18F]FClO_3_ (Adam et al. 1986)” 🡪 “with [^18^F]FClO_3_ (Hiller et al. 2008)”

“or [^18^F]XeF_2_ (Chirakal et al. 1984; Oberdorfer et al. 1988)” 🡪 “or [^18^F]XeF_2_ (Adam et al. 1986; Firnau et al. 1980)”

“Oberdorfer (Teare et al. 2010)” 🡪”Oberdorfer (Oberdorfer et al. 1988)”

“([18F]NFSI) (Teare et al. 2010)” 🡪 “([^18^F]NFSI) (Teare et al. 2007)”

*“bis*(triflate)) (Buckingham et al. 2015a)” 🡪 *“bis*(triflate)) (Teare et al. 2010)”

“[^18^F]N-fluoropyridinium triflate (Teare et al. 2010).” 🡪 “[^18^F]N-fluoropyridinium triflate (Oberdorfer et al. 1988).”

“enol ethers (Teare et al. 2010),” 🡪 “enol ethers (Teare et al. 2007),”

“aldehydes (Nodwell et al. 2017)” 🡪 “aldehydes (Buckingham et al. 2015)”

“tetrapeptides (Fig. 1C) (Yuan et al. 2018; Chaly et al. 1994).” 🡪 “tetrapeptides (Fig. 1C) (Nodwell et al. 2017;Yuan et al. 2018).”

“[18F]AcOF (Namavari et al. 1992),” 🡪 “[^18^F]AcOF (Chaly et al. 1994),”

“(Fig. 1D) (Stenhagen et al. 2013).” 🡪 “(Fig. 1D) (Namavari et al. 1992).”

“(Fig. 1D) (Liu et al. 2016).” 🡪 “(Fig. 1D) (Stenhagen et al. 2013).”

“click chemistry (Hefter and McLay 1988).” 🡪 “click chemistry (Liu et al. 2016).”

**18F‑Fluorination with [18F]fluoride**

“transformations (Mossine et al. 2017)” 🡪 “transformations (Hefter et al. 1988).”

“quarternary” 🡪 “quaternary”

“drying (O’Hagan et al. 2002).” 🡪 “drying (Mossine et al. 2017)”

“enzyme (Kuchar and Mamat 2015).” 🡪 “enzyme (O’Hagan et al. 2002).”

**Csp2–F bond formation**

“radiotracers (Yerien et al. 2016).” 🡪 “radiotracers (Kuchar et al. 2015).”

“methods for (Hiller et al. 2018) F” 🡪 “methods for ^19^F”

“for (Hiller et al. 2018) F-fluorination” 🡪 “for ^19^F-fluorination”

“between (Hiller et al. 2018) F- and 18F-chemistry” 🡪 “between ^19^F- and ^18^F-chemistry”

***Metal‑free Csp2–F bond formation***

“Balz-Schiemann reaction (Fig. 3A) (Knochel and Zwernemann 1996).” 🡪 “Balz-Schiemann reaction (Fig. 3A) (Balz et al. 1927).”

“results (Argentini et al. 1994).” 🡪 “results (Knochel et al. 1996).”

“5-[^18^F]FDOPA (Pages et al. 2001)” 🡪 “5-[^18^F]FDOPA (Argentini et al. 1994)”

“methodology (Finger and Kruse 1956).” 🡪 “methodology (Pages et al. 2001).”

“arenes (Preshlock et al. 2016a)” 🡪 “arenes (Finger and Kruse 1956).”

“with [18F]F− (Lazarova et al. 2007).” 🡪 “with [^18^F]F− (Preshlock et al. 2016).”

“SNAr conditions (Fig. 3C) (Narayanam et al. 2017).” 🡪 “SNAr conditions (Fig. 3C) (Lazarova et al. 2007).”

“via SNAr (Grushin et al. 1992).” 🡪 “via SNAr (Narayanam et al. 2017).”

“diaryliodonium salts (Pike and Aigbirhio 1955)” 🡪 “diaryliodonium salts (Grushin et al. 1992),”

“(Fig. 3E) (Yamada et al. 1974).” 🡪 “(Fig. 3E) (Pike and Aigbirhio et al. 1995).”

*“ipso*-substitution (Ross et al. 2007).” 🡪 “*ipso*-substitution (Yamada et al. 1974).”

“(Fig. 3F) (Satyamurthy and Barrio 2010).” 🡪 “(Fig. 3F) (Ross et al. 2007).”

“Barrio (Rotstein et al. 2014).” 🡪 “Barrio (Satyamurthy and Barrio 2010),”

“Liang and Vadev” 🡪 “Liang and Vasdev”

“(Fig. 3G) (Liang et al. 2019).” 🡪” (Fig. 3G) (Rotstein et al. 2014).”

“GBq/μmol) (Szpera et al. 2020).” 🡪 “GBq/μmol) (Liang et al. 2019).”

“C–H 18F-fluorination (Neumann et al. 2016)” 🡪 “C-H ^18^F-fluorination (Szpera et al. 2019)”

“PhenoFluor-mediated (Hiller et al. 2018) F” 🡪 “PhenoFluor-mediated ^19^F”

“phenols (Schimler et al. 2017).” 🡪 “phenols (Neumann et al. 2016).”

“2017 (Fig. 4B) (Meada et al. 1987).” 🡪 2017 (Fig. 4B) (Schimler et al. 2017).”

“limited by Hiller et al. (2018) F-fluoride” 🡪 “limited by ^19^F-fluoride”

“1987 (Gendron et al. 2018)” 🡪 “1987 (Meada et al. 1987).”

“(Fig. 4C) (Xu et al. 2020).” 🡪 “(Fig. 4C) (Gendron et al. 2018).”

“respectively (Xu et al. 2020).” 🡪”respectively (Gendron et al. 2018).”

“(Fig. 4C) (Simons 1949).” 🡪 “(Fig. 4C) (Xu et al. 2020).”

“by Simons and Harland (1949)” 🡪 “by Simons and Harland 1949;”

“(voltage) (Sawamura et al. 2012).” 🡪 “(voltage) (Simons et al. 1949).”

“developed (Rozhkov and Alyev 1975).” 🡪 “were developed (Sawamura et al. 2012).”

“Rozhkov (Knunyants et al. 1970), Knunyants (Narizuka and Fuchigami 1993) and

Fuchigami (Reischl et al. 2002) electrochemical 18F-fluorination was first reported

by Reischl (2002), Kienzle et al. (2005). “ 🡪 “ Rozhkov (Rozhkov and Alyev 1975), Knunyants (Knunyants et al. 1970) and Fuchigami (Narizuka and Fuchigami 1993) electrochemical 18F-fluorination was first reported by Reischl (Reischl et al. 2002).”

“1.5–2 V (He et al. 2001).” 🡪”1.5–2 V (Kienzle et al. 2005).”

“(Am = 0.043 GBq/μmol) (Lebedev et al. 2017).” 🡪 “(Am = 0.043 GBq/μ mol) (He et al. 2001).”

“0.111 GBq/μmol) (Gao et al. 2012).” 🡪 ”0.111 GBq/μ mol) (Lebedev et al. 2017).”

“(Fig. 4E) (Buckingham et al. 2015b).” 🡪 “(Fig. 4E) (Gao et al. 2012).”

“sulfonamides.(Chen et al. 2019)” 🡪 ”sulphonamides (Buckingham et al. 2015).”

“(Fig. 4E) (Wang et al. 2020).” 🡪 “(Fig. 4E) (Chen et al. 2019).”

“(TBPA) (Douglas and Nicewicz 2019).” 🡪 ”(TBPA) (Wang et al. 2020).”

“nucleofuges.(Tay et al. 2020)” 🡪 “nucleofuges (Douglas and Nicewicz 2019).”

“GBq/μmol.(Watson et al. 2009).” 🡪 “GBq/μmol (Tay et al. 2020).”

***Metal‑mediated/catalysed Csp*2*–F bond formation***

“fluoride sources. (Lazarova et al. 2007).” 🡪 “fluoride sources (Preshlock et al. 2016).”

species.(Cardinale et al. 2012) 🡪species (Watson et al. 2009).

**From Cardinale et al. 2012 the references are shifted by one (see the examples below)**

“(Fig. 5A) (Hull et al. 2006).” 🡪 “(Fig. 5A) (Cardinale et al. 2012).”

“(Fig. 5A) (Boursalian and Ritter 2018).” 🡪 “(Fig. 5A) (Hull et al. 2006).”

“complexes.(Lee et al. 2011)” 🡪”complexes (Boursalian and Ritter 2018).”

“[18F]fluoroarenes.(Lee et al. 2012)” 🡪 “[^18^F]fluoroarenes (Lee et al. 2011).”

“(Fig. 5B) (Zlatopolskiy et al. 2015).” 🡪 “(Fig. 5B) (Lee et al. 2012).”

“respectively.(Casitas et al. 2011).” 🡪”respectively (Zlatopolskiy et al. 2015).”

“Ribas(Fier and Hartwig 2012) and Hartwig,(Ye et al. 2013)” 🡪 “Ribas (Casitas

et al. 2011) and Hartwig (Fier et al. 2011)”

“Cu(OTf)2 (Ye et al. 2013).” 🡪”Cu(OTf)2.(Tredwell et al. 2014)”

“(Fig. 5C) (Preshlock et al. 2016b).” 🡪 “(Fig. 5C) (Tredwell et al. 2014).”

“[18F]FDA,(Taylor et al. 2017)” 🡪 “[18F]FDA (Preshlock et al. 2016),”

“heteroarenes.(Guibbal et al. 2020)” 🡪 “heteroarenes (Taylor et al. 2017).”

“18F-fluorodeboronation.(Makaravage et al. 2016)” 🡪 “18F-fluorodeboronation (Guibbal et al. 2020).”

“radiofluorination,(Mossine et al. 2019)” 🡪”radiofluorination (Makaravage et al. 2016),”

“imaging.(Mossine et al. 2020; Ichiishi et al. 2014)” 🡪”imaging (Mossine et al. 2019, 2020).”

“bromides.(Wright et al. 2021)” 🡪”bromides(Ichiishi et al. 2014).”

“(hetero)arenes.(Mu et al. 2014)” 🡪 “(hetero)arenes (Wright et al. 2021).”

“Liu (Sharninghausen et al. 2020)” 🡪”Liu (Mu et al. 2014),”

“(Fig. 5C) (Xu et al. 2021).” 🡪 “(Fig. 5C) (Sharninghausen et al. 2020).”

“nucleophilic (Hiller et al. 2018) 🡪F-fluorination nucleophilic ^19^F-fluorination”

“by Ritter (2021) and by MacMillan (2014) 🡪by Ritter (Xu et al. 2021) and by MacMillan (Chen et al. 2021);”

“reported (O’Hagan et al. 2002; Bowden et al. ). 🡪 reported (Mossine et al. 2017; Richarz et al. 2014).”

“RCYs (O’Hagan et al. 2002). 🡪 RCYs (Mossine et al. 2017).”

“step (O’Hagan et al. 2002). 🡪step (Mossine et al. 2017).”

“[18F]olaparib (Sergeev et al. 2015). 🡪 [18F]olaparib (Bowden et al. 2021).”

**From Sergeev et al. 2012 the references are shifted by one (see the examples below)**

agent (Wu 2014). 🡪agent (Sergeev et al. 2015).

***Csp3–F bond formation***

“reactions (Liang et al. 2017).” 🡪”reactions (Wu et al. 2014).”

“nucleophilicity (Hamacher et al. 1986),” 🡪”nucleophilicity (Liang et al. 2017),”

“water (Kuchar and Mamat 2015).” 🡪 “water (O’Hagan et al. 2002).”

“(Fig. 6A) (Kim et al. 2004).” 🡪 “(Fig. 6A) (Hamacher et al. 1986).”

“process (Moon et al. 2006). 🡪”process (Kim et al. 2004)”

“respectively (Kamarainen et al. 2004).” 🡪”respectively (Moon et al. 2006).”

“(hypoxia) (Morgan et al. 2021). *cis*-4-[18F]fluoro-L-proline (collagen) (Wester et al. 1999) and *O*-(2-[18F]

fluoroethyl)-L-tyrosine (glioma grading) (Singh and Shreeve 2002)” 🡪 “(hypoxia) (Kamarainen et al. 2004), cis-4-[18F]fluoro-L-proline (collagen) (Morgan et al. 2021) and O-(2-[18F] fluoroethyl)-L-tyrosine (glioma grading) (Wester et al. 1999)”

“(Deoxo-Fluor) (Straatmann and Welch 1977).” 🡪 “(Deoxo-Fluor) (Singh et al. 2002).”

“Welch in 1977 (Nielsen et al. 2015)”🡪”Welch in 1977 (Straatmann et al. 1977).”

“(PyFluor) (Goldberg et al. 2016)”🡪 “(PyFluor) (Nielsen et al. 2015)”

“(AlkylFluor) (Tarantino and Hammond 2020)”🡪 “(AlkylFluor)(Goldberg et al. 2016)”

“(Fig. 6B) (Goldberg et al. 2016).” 🡪”(Fig. 6B) (Nielsen et al. 2015).”

“triflate (Testa et al. 2019).” 🡪 ”triflate (Tarantino and Hammond 2020).”

“pathways (Hintermann et al. 2006).” 🡪 “pathways (Testa et al. 2019).”

“Togni (Hazari et al. 2009) Brown (Hollingworth et al. 2011) Gouverneur (Hollingworth et al. 2011; Katcher and Doyle 2010) Doyle (Topczewski et al. 2011) and Nguyen (Mixdorf et al. 2019) 🡪 Togni (Hintermann et al. 2006), Brown (Hazari et al. 2009), Gouverneur (Hazari et al. 2009; Hollingworth et al. 2011), Doyle (Katcher et al. 2010) and Nguyen (Topczewski et al. 2011)”

“52% (Fig. 6C) (Hollingworth et al. 2011).” 🡪”52% (Fig. 6C) (Hazari et al. 2009).”

“situ (Fig. 6C) (Mixdorf et al. 2019).” 🡪 ”in situ (Fig. 6C) (Topczewski et al. 2011).”

“37% (Benedetto et al. 2013).” 🡪 “37% (Mixdorf et al. 2019).”

“76% (Fig. 6C) (Bruns and Haufe 2000).” 🡪 “76% (Fig. 6C) (Benedetto et al. 2013).”

“Bruns and Haufe (2014).” 🡪 “Bruns and Haufe (Bruns et al. 2000),”

“moiety (Verhoog et al. 2019).” 🡪 “moiety (Graham et al. 2014).”

“(CH3CO2H, CH3SO3H) (Huang et al. 2014).” 🡪” (CH3CO2H, CH3SO3H) (Verhoog et al. 2019).”

“PhIO (Fig. 6E) (Liu et al. 2018).” 🡪 “PhIO (Fig. 6 E) (Huang et al. 2014).”

“Precursors (Huang et al. 2015).” 🡪”precursors (Liu et al. 2018).”

“18F-fluorination (Carroll et al. 2015).” 🡪 “18F-fluorination (Huang et al. 2015).”

“AgOTf (Gray et al. 2016).” 🡪 “AgOTf (Carroll et al. 2015).”

“2016 (Fig. 6F) (Gonzalez et al. 2018).” 🡪 “2016 (Fig. 6F)(Gray et al. 2016).”

“fluoride (Kohlhepp and Gulder 2016).” 🡪 “fluoride (Gonzalez et al. 2018)”

“In Hiller et al. (2018) F-mode 🡪 In 19F-mode,”

“fluorobenziodoxole (Gonzalez et al. 2019)” 🡪 “fluorobenziodoxole (Kohlhepp et al. 2016)”

“(Fig. 6F) (Geary et al. 2015).” 🡪 “(Fig. 6F) (Gonzalez et al. 2019).”

“fluorobenziodoxole (Yang et al. 2019).” 🡪 “fluorobenziodoxole (Geary et al. 2015),”

“[18F]Bu4NF (Webb et al.2020).” 🡪 “[18F]Bu4NF (Yang et al. 2017).”

“(up to 396 GBq/μmol) (Kohlhepp and Gulder 2016).” 🡪 “(up to 396 GBq/μmol) (Gonzalez

et al. 2018).”

“(Fig. 6H) (Waldmann et al. 2017).” 🡪 “(Fig. 6H) (Webb et al. 2020).”

“(e.g. Et3N·3HF) (Balandeh et al. 2018). In 20^18”^ 🡪”(e.g. Et3N·3HF) (Waldmann et al. 2017). In 2018”

“(pH = 3) (Barata-Vallejo et al. 2015).” 🡪 “(pH = 3)(Balandeh et al. 2018).”

“pace (Prchalova et al. 2014)” 🡪 “pace (Barata-Vallejo et al. 2015),”

“demonstrated (Pan 2019).” 🡪 “demonstrated (Prchalova et al. 2014).”

“demand (Ido et al. 1979)” 🡪 “demand (Pan 2019)”

“exchange (Fig. 7A) (Angelini et al. 1990).” 🡪 “exchange (Fig. 7A) (Ido et al. 1979).”

“(18-crown-6)” 🡪 “(18-crown-6)”

“(~ 50% RCY) (Fig. 7A) (Kilbourn et al. 1990).” 🡪”(~ 50% RCY) (Fig. 7A) (Angelini et al. 1990).”

“by Kilbourn (Fig. 7A)(Hammadi and Crouzel 1993). “🡪” by Kilbourn (Fig. 7A) (Kilbourn et al. 1990).”

“GBq/μmol) (Prabhakaran et al. 2007).” 🡪 ”GBq/μmol) (Hammadi et al. 1993).”

“GBq/μmol (Verhoog et al. 2016).” 🡪”GBq/μmol (Prabhakaran et al. 2007).”

“(0.03 GBq/μmol) (Johnstrom and Stone-Elander 1995).” 🡪 “(0.03 GBq/μmol) (Verhoog et al. 2016).”

“[18F]F− (Suehiro et al. 2011).” 🡪 “[^18^F]F− (Johnstrom and Stone-Elander 1995).”

“(Fig. 7C) (Riss et al. 2011).” 🡪 “(Fig. 7C) (Suehiro et al. 2011).”

“pathology (Riss and Aigbirhio 2011).” 🡪”pathology (Riss et al. 2013).”

“improvements (Kramer et al. 2020)”🡪 “improvements (Riss and Aigbirhio 2011).”

“imaging (Frost et al. 2019). 🡪 “imaging (Kramer et al. 2020).”

“(Fig. 7D) (Gomez et al. 2016).” 🡪 “(Fig. 7D) (Frost et al. 2019).”

“Schou (Fig. 7E) (Josse et al. 2011).” 🡪 “Schou (Fig. 7E) (Gomez et al. 2016).”

“dithioates (Fig. 7F) (Dolbier et al. 2001). 🡪 dithiolates (Fig. 7F) (Josse et al. 2011).”

“RCY and Am (Eskola et al. 2012; Prakash et al. 2003). F-Radiolabelling” 🡪 “RCY and A_m_ (Dolbier et al. 2001) ^18^F-Radiolabelling”

“GBq/μmol) (Yin et al. 2012).” 🡪 “GBq/μmol) (Prakash et al. 2003).”

“carboxylic acids (Mizuta et al. 2013).” 🡪 carboxylic acids (Yin et al. 2012),”

“(Fig. 7H) (MacNeil and Burton 1991).” 🡪 “(Fig. 7H) (Mizuta et al. 2013).”

“work of Burton (1993) and Chen (2013)” 🡪 “work of Burton (MacNeil et al. 1991) and Chen (Duan et al.

1993),”

“(Fig. 7I) (Born et al. 2014).” 🡪 “(Fig. 7I) (Huiban et al. 2013).”

“[^18^F]CF_3_H (Ruhl et al. 2014).” 🡪 “ [^18^F]CF_3_H (Born et al. 2014).”

“(DIPEA) (Fig. 7I) (Ivashkin et al. 2014).” 🡪 “(DIPEA) (Fig. 7I) (Rühl et al. 2014).”

“(Fig. 7I) (Szpera et al. 2020).” 🡪 “(Fig. 7I) (Ivashkin et al. 2014).”

“(Fig. 7J) (Pees et al. 2021).” 🡪 “(Fig. 7J) (Pees et al. 2021).”

“seminal studies (Liu et al. 2018; Huang et al. 2015; Carroll et al. 2015).” 🡪 “seminal studies (Liu et al. 2018; Huang et al. 2015; Huang et al. 2014).”

“(Fig. 7K) (Gray et al. 2016).” 🡪 “(Fig. 7K) (Carrol et al. 2015).”

“13 GBq/μmol) (Fig. 7L) (Yang et al. 2019; Pees et al. 2020; Levin et al. 2017).” 🡪 “13 GBq/μmol) (Fig. 7L) (Pees et al. 2021, 2020; Yang et al. 2019).”

“(Fig. 7M) (Zafrani et al. 2019)” 🡪 “(Fig. 7M) (Levin et al. 2017).”

***Radiosynthesis of [*18*F]difluoromethyl‑containing molecules***

“agrochemicals (Sap et al. 2021; Shi et al. 2016).” 🡪 “agrochemicals (Zafrani et al. 2019; Sap et al. 2021).”

“18F-source (Born et al. 2014). “ 🡪 “18F-source (Huiban et al. 2013).”

**“**(Fig. 8B) (Johnstrom and Stone-Elander 1995)” 🡪 “ (Fig. 8B) (Verhoog et al. 2016)”

“(Fig. 8C) (Yuan et al. 2017).” 🡪 “(Fig. 8C) (Shi et al. 2016).”

“(Fig. 8D) (Sap et al. 2019).” 🡪 “(Fig. 8D) (Yuan et al. 2017)”

“(Fig. 8E) (Trump et al. 2019).” 🡪 “(Fig. 8E) (Sap et al. 2019).”

“(Fig. 8F) (Rong et al. 2016).” 🡪 “(Fig. 8F) (Trump et al. 2019).”

“(Hu reagent) (Trump et al. 2020),” 🡪 “(Hu reagent) (Rong et al. 2016),”

“platform (Landelle et al. 2014).” 🡪 “platform (Trump et al. 2020).”

***Radiosynthesis of [*18*F]SCF*3*, SCHF*2*,OCF*3 *and OCHF*2*‑containing molecules***

“lipophilicity (Trump et al. 2020).” 🡪 “lipophilicity (Landelle et al. 2014).”

“sclerosis (Silverstone et al. 1979)” 🡪 “sclerosis (Bellingham et al. 2011)”

“tiflorex (Khotavivattana et al. 2015)” 🡪 “tiflorex (Silverstone et al. 1979),”

“exchange (Zheng et al. 2015).” 🡪 “exchange (Khotavivattana et al. 2015).”

“(Fig. 9B) (Zheng et al. 2017).” 🡪 “(Fig. 9B) (Zheng et al. 2015).”

“derivatives (Carbonnel et al. 2017)” 🡪 “derivatives (Zheng et al. 2017).”

“(Fig. 9B) (Eskola et al. 2012; Wu et al. 2019). F-Labelled” 🡪 “(Fig. 9B) (Carbonnel et al. 2017). ^18^F-Labelled”

“(Fig. 9C) (Zhao et al. 2020).” 🡪 “(Fig. 9C) (Wu et al. 2019).”

“(Fig. 9D) (Milcent and Crousse 2018).” 🡪 “(Fig. 9D) (Zhao et al. 2020).”

“N-CF3(Gaba and Mohan 2016) and N-CF2H (Scattolin et al. 2019)” 🡪 “N-CF_3_ (Milcent et al. 2018) and N-CF_2_H (Gaba et al. 2016),”

“reagent (Andre/s et al. 2014)” 🡪 “reagent (Scattolin et al. 2019)”

“nitrogen (Bernard-Gauthier et al. 2018).” 🡪 “nitrogen (Andre´s et al. 2014).”

**18F‑Labelling of biomolecules via 18F–C bond construction**

“elsewhere (Schirrmacher et al. 2017).” 🡪 “elsewhere (Bernard-Gauthier et al. 2018).”

“incorporation (Vaidyanathan and Zalutsky 1992).” 🡪 “incorporation (Schirrmacher et al. 2017).”

“([18F]SFB) (Marik and Sutcliffe 2007) 4-[18F]fluorobenzoic acid ([18F]FBA) (Li et al. 2008) N-[6-(4-[18F]fluorobenzylidene)aminooxyhexyl]maleimide ([18F]FBAM) (Kiesewetter et al. 2011) and *N*-[2-(4-[18F]fluorobenzamido)ethyl]maleimide ([18F]FBEM) (Li et al. 2011)” 🡪 “([^18^F]SFB) (Vaidyanathan et al. 1992),193 4-[^18^F]fluorobenzoic acid ([^18^F]FBA) (Marik et al. 2007), N-[6-(4-18F]fluorobenzylidene)aminooxyhexyl] maleimide ([^18^F]FBAM) (Li et al. 2008) and *N*-[2-(4-^18^F]fluorobenzamido)ethyl]maleimide ([^18^F]FBEM) (Kiesewetter et al. 2011)”

“heterocycles (Gouverneur 2011; Becaud et al. 2009).” 🡪 “heterocycles (Gouverneur, 2011) (Li et al. 2011).”

“(Fig. 10B) (Rickmeier and Ritter 2018).” 🡪 “(Fig. 10B) (Becaud et al. 2009).”

“(details illustrated in Fig. 1C) (Yuan et al. 2018; Chaly et al. 1994)” 🡪 “(details illustrated in Fig. 1C) (Nodwell et al. 2017; Yuan et al. 2018),”

“(Fig. 10C) (Verhoog et al. 2018). In 20^18^,” 🡪 “(Fig. 10C) (Rickmeier et al. 2018). In 2018,”

“(Fig. 10D) (Gao et al. 2013).” 🡪 “(Fig. 10D) (Verhoog et al. 2018).”

“Davis (Ichiishi et al. 2018) and of Krska (Kee et al. 2020)” 🡪 “Davis(Gao et al. 2013) and of Krska (Ichiishi et al. 2018),”

“(Fig. 10E) (O′Hagan et al. 2002).” 🡪 “(Fig. 10E) (Kee et al. 2020).”

“(Fig. 10F) (Thompson et al. 2015).” 🡪 “(Fig. 10F) (O’Hagan et al. 2002).”

“95% (Lowe et al. 2019).” 🡪 “95% (Thompson et al. 2015).”

“(AdoSeMet) (Pupo et al. 2018).” 🡪 “(AdoSeMet) (Lowe et al. 2019).”

“motifs (Lowe et al. 2019).” 🡪 “motifs (Thompson et al. 2015).”

“reactivity (Pupo et al. 2019; Roagna et al. 2020; Ibba et al. 2020).” 🡪”reactivity

(Pupo et al. 2018, 2019; Roagna et al. 2020; Ibba et al. 2020).”
